# New Triazinoindole Bearing Benzimidazole/Benzoxazole Hybrids Analogs as Potent Inhibitors of Urease: Synthesis, In Vitro Analysis and Molecular Docking Studies

**DOI:** 10.3390/molecules27196580

**Published:** 2022-10-04

**Authors:** Sundas Mumtaz, Shahid Iqbal, Mazloom Shah, Rafaqat Hussain, Fazal Rahim, Wajid Rehman, Shoaib Khan, Obaid-ur-Rahman Abid, Liaqat Rasheed, Ayed A. Dera, Hanan A. Al-ghulikah, Sana Kehili, Eslam B. Elkaeed, Hamad Alrbyawi, Mohammed Issa Alahmdi

**Affiliations:** 1 Department of Chemistry, Hazara University, Mansehra 21300, Pakistan; 2 Department of Chemistry, School of Natural Sciences (SNS), National University of Sciences and Technology (NUST), H-12, Islamabad 46000, Pakistan; 3 Department of Chemistry, Abbottabad University of Science and Technology (AUST), Abbottabad 22010, Pakistan; 4 Department of Clinical Laboratory Sciences, College of Applied Medical Sciences, King Khalid University, Abha 61413, Saudi Arabia; 5 Department of Chemistry, College of Science, Princess Nourah bint Abdulrahman University, P.O. Box 84428, Riyadh 11671, Saudi Arabia; 6 Adham University College, Umm Al-Qura University, Makkah 21955, Saudi Arabia; 7 Department of Pharmaceutical Organic Chemistry, Faculty of Pharmacy (Boys), Al-Azhar University, Cairo 11884, Egypt; 8 Department of Pharmaceutical Sciences, College of Pharmacy, AlMaarefa University, Riyadh 13713, Saudi Arabia; 9 Pharmaceutics and Pharmaceutical Technology Department, College of Pharmacy, Taibah University, Medina 42353, Saudi Arabia; 10 Department of Chemistry, Faculty of Science, University of Tabuk, Tabuk 71491, Saudi Arabia

**Keywords:** synthesis, triazinoindole, benzimidazole, benzoxazole, urease, structure-activity relationship and molecular docking

## Abstract

Twenty-four analogs based on triazinoindole bearing benzimidazole/benzoxazole moieties (**1**–**25**) were synthesized. Utilizing a variety of spectroscopic methods, including ^1^H-, ^13^C-NMR, and HREI-MS, the newly afforded compounds (**1**–**25**) were analyzed. The synthesized analogs were tested against urease enzyme (in vitro) as compared to the standard thiourea drug. All triazinoindole-based benzimidazole/benzoxazole analogs (**1**–**25**) exhibited moderate to excellent inhibition profiles, having IC_50_ values of 0.20 ± 0.01 to 36.20 ± 0.70 μM when evaluated under the positive control of thiourea as a standard drug. To better understand the structure–activity relationship, the synthesized compounds were split into two groups, “A” and “B.” Among category “A” analogs, analogs **8** (bearing tri-hydroxy substitutions at the 2,4,6-position of aryl ring C) and **5** (bearing di-hydroxy substitutions at the 3,4-position of aryl ring C) emerged as the most potent inhibitors of urease enzyme and displayed many times more potency than a standard thiourea drug. Besides that, analog **22** (which holds di-hydroxy substitutions at the 2,3-position of the aryl ring) and analog **23** (bearing *ortho*-fluoro substitution) showed ten-fold-enhanced inhibitory potential compared to standard thiourea among category “B” analogs. Molecular docking studies on the active analogs of each category were performed; the results obtained revealed that the presence of hydroxy and fluoro-substitutions on different positions of aryl ring C play a pivotal role in binding interactions with the active site of the targeted urease enzyme.

## 1. Introduction

Urease is a nickel-dependent metalloenzyme that catalyzes the hydrolytic degradation of urea to afford carbon dioxide and ammonia [1]. The survival of *Helicobacter pylori* was enhanced by liberating ammonia via increasing the value of pH in an aqueous solution [2]. Moreover, it was noted that numerous disorders related to gastroduodenal function were caused by *Helicobacter pylori*. These disorders include urinary catheter encrustation, gastric cancer, duodenal ulcers, and peptic ulcers [3,4]. Urea breakdown is controlled by urease inhibitors, which also prevent ureolytic bacterial infections that lead to destruction [5]. Keeping in mind the biological significance of organic heterocyclic compounds, which function as inhibitors against urease enzymes, medicinal chemists have paid substantial attention to synthesizing novel heterocyclic urease inhibitors as possible drug candidates [6]. Numerous heterocyclic substances have been used to inhibit the urea enzyme for the treatment of gastroduodenal disorders over the past few decades, including the derivatives of hydroxamic acid and urea, triazole, coumarin, Schiff bases, semicarbazone, oxadiazole, and piperidine [7,8,9,10,11,12,13,14,15]. Furthermore, a variety of antifreeze agents have been reported and have, therefore, gained significant attention from medicinal chemists by inhibiting urease enzymes [16].

Triazinoindole scaffolds were reported to have numerous biological activities, such as anti-depressants [17], antihypertensives [18], antihypoxic [19], anti-inflammatory [20], and antibacterial and antifungal profiles [21]. Furthermore, some triazinoindole analogs have been identified as active drugs for the treatment of the common cold [22,23,24,25].

Benzimidazole scaffolds showed a broad range of biological activities, including anti-glycation [26], anti-tumor [27], anti-diabetic [28], antiprotozoal [29], anti-psychotic [30], ant-viral [31] and antioxidant profiles [32]. There are several anticancer drugs (preclinical or clinical trials) with a benzimidazole moiety in their core skeleton, including nocodazole [33], bendamustine [34], and Hoechst 33,258 [35].

In several therapeutic fields, such as antifungal [36], antibacterial [37], anti-inflammatory [38], and anticancer [39] contexts, benzoxazole derivatives have been identified as “privileged scaffolds” to access compounds with intriguing biological characteristics. Furthermore, boxazomycin B, nakijinol B, benoxaprofen, boxazomycin A, calcimycin, nataxazole, nocarbenzoxazole G, flunoxaprofen, and chloroxazole are biologically active drugs that hold a benzoxazole skeleton in their core structure [40,41,42].

**Figure 1:**
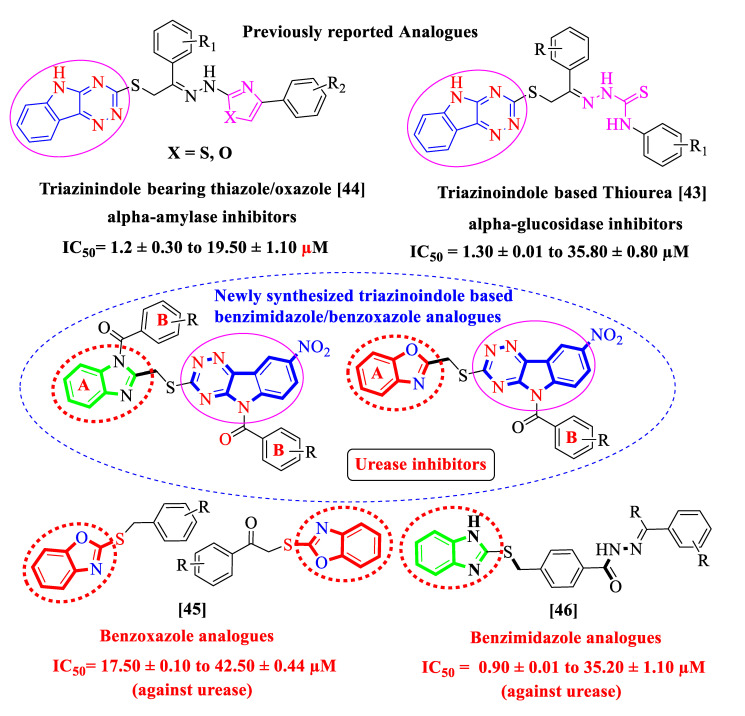
Rationale of the current study.

Encouraged by the success of nitrogen-, oxygen-, and sulfur-containing fused heterocyclic compounds, such as triazinoindole [43,44], benzoxazole [45], and benzimidazole [46] as potent inhibitors of alpha-amylase, alpha-glucosidase, and the urease enzyme, we developed a method for the synthesis of hybrid analogs, based on triazinoindole bearing benzimidazole/benzoxazole (**1**–**25**) scaffolds in an effort to further investigate the inhibitory potential, with the hopes that it could demonstrate more effectiveness against the urease enzyme (Figure 1).

Our research group has been working on studying alpha amylase and alpha glucosidase, as well as urease, which are also included in the targeted study [47,48].

## 2. Results and Discussion

### 2.1. Chemistry

#### 2.1.1. Synthesis of 7-Nitro-5H-[1,2,4]triazino[5,6-b]indole-3-thiol (**III**)

Thiosemicarbazide (**II**) is a reacted isatin solution (**I**) that is stirred in water, along with potassium carbonate (as a catalyst). The resultant mixture was held under reflux and stirred until the conversion was complete (monitored by TLC). Triazinoindole-3-thiol (**III**), which was obtained after the solvent evaporated while being agitated at refluxing temperature, was further purified after being washed with n-hexane, to provide a pure form of the intermediate 3-marcapto triazinoindole (**IV**).

#### 2.1.2. Synthesis of 2-(Chloromethyl)-1H-benzo[d]imidazole (**VIb**)

The intermediate 2-(chloromethyl)-1H-benzo[d]imidazole (**IVb**) was synthesized through the cyclization of phenyl 1,2-diamine (**IVa**) in a 4N HCl solution under reflux conditions.

#### 2.1.3. Synthesis of 2-(Chloromethyl)benzo[d]oxazole (**VIb**)

In the formation of intermediates (**VIb**), the stirred solution of 2-aminophenol (**VIa**) in 4N HCl solution underwent cyclization via stirring at the refluxing temperature over a pre-heated sand bath to access the formation of benzoxazole-based intermediate (**VIb**). After being stirred at the refluxing temperature, the solvent was evaporated by applying conditions of reduced pressure to yield the solid form of 2-(chloromethyl) benzo[d]oxazole (**VIb**).

#### 2.1.4. Synthesis of 3-(((1H-Benzo[d]imidazol-2-yl)methyl)thio)-8-nitro-5H-[1,2,4]triazino[5,6-b]indole (**V**)

The following phase was the sequential addition of an intermediate (**IVb**) and triethylamine in ethanol, followed by the direct dehydrogenation of an intermediate (**III**). To enable the synthesis of a benzimidazole intermediate for triazinoindole, the crude mixture was stirred continuously until the consumption of both intermediates (**III**) and (**IVb**) was completed, which yielded compound (**V**).

#### 2.1.5. Synthesis of 2-(((8-Nitro-5H-[1,2,4]triazino[5,6-b]indol-3-yl)thio)methyl)benzo[d]oxazole (**VI**)

Additionally, a triazinoindole-based benzoxazole intermediate was produced by treating an intermediate (**III**) with an intermediate (**VIb**) in ethanol and then adding the necessary quantity of triethylamine, which yielded compound (**VI**).

#### 2.1.6. Synthesis of Substituted 2-(((8-Nitro-5H-[1,2,4]triazino[5,6-b]indol-3-yl)thio)methyl)benzo[d]oxazole (**1**–**12**)

**Scheme 1:**
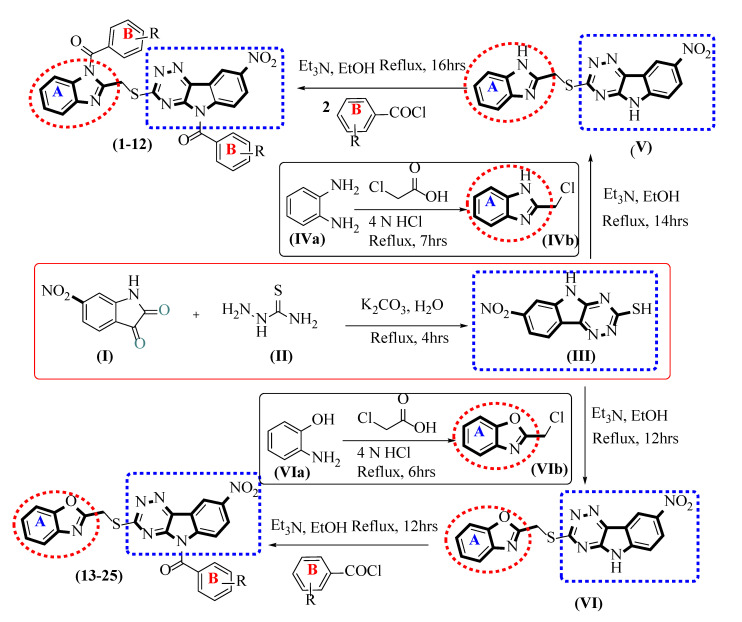
Synthesis of triazinoindole-based benzimidazole/benzoxazole derivatives.

Encouraged by the success of the triazinoindole-based benzimidazole (**V**) synthesis, we sought to broaden the application of this practical approach by treating the triazinoindole-based benzimidazole (**V**) with various substituted benzoyl chlorides in ethanol, under the catalytic action of triethylamine (Scheme 1). We were pleased to find that the resulting residue was stirred and refluxed at refluxing temperature to afford the synthesis of the desired triazinoindole bearing benzimidazole (**1**–**12**).

#### 2.1.7. Synthesis of Substituted (3-((Benzo[d]oxazol-2-ylmethyl)thio)-8-nitro-5H-[1,2,4]triazino[5,6-b]indol-5-yl)(phenyl)methanone (**13**–**25**)

As with benzoxazole (**VI**), we sought to broaden the application of this practical approach by treating the triazinoindole-based benzoxazole (**VI**) with various substituted benzoyl chlorides in ethanol, under the catalytic action of triethylamine. We were pleased to find that the resulting residue was stirred and refluxed at the refluxing temperature to afford the synthesis of the desired triazinoindole bearing benzoxazole (**13**–**25**).

### 2.2. In Vitro Urease Inhibitory Activity

All newly afforded triazinoindole bearing benzimidazole/benzoxazole derivatives (**1**–**25**) were tested for their ability to inhibit the urease enzyme (in vitro). It is noteworthy that, in comparison to the thiourea standard, all triazinoindole-containing benzimidazole/benzoxazole compounds exhibited moderate to excellent inhibitory activity against the urease enzyme (Table 1). The synthesized compounds were divided into two groups, in order to better understand the structure-activity relationship (SAR). When benzotriazole reacts with various substituted benzoic acids that have an amide bond, 12 derivatives (**1**–**12**) of “Category A” are produced, whereas the 12 derivatives (**13**–**25**) of “Category B” are also produced when 3-marcaptotriazinoindole substrate reacts with benzimidazole, which has an active methylene part with the chloro group (Table 1). The triazinoindole, benzimidazole, benzoxazole, carbonyl group, and aryl part/s of the structure are all actively contributing to the activity, and any variations in this activity can be attributed to the various substitution groups on the aryl ring/s (R), according to the limited structure-activity relationship (SAR).

#### 2.2.1. Structure-Activity Relationship (SAR) for Urease Inhibitory Activity

Based on variable replaced “R” groupings, the restricted structure-activity relationship for each category was examined. Even if the triazinoindole and carbonyl moieties are playing a key role in the SAR for alpha-amylase inhibitory activity in both categories, the variations in these activities are brought on by the varying features of the aryl ring/s (R).

#### 2.2.2. Category “A”

**Figure 2:**
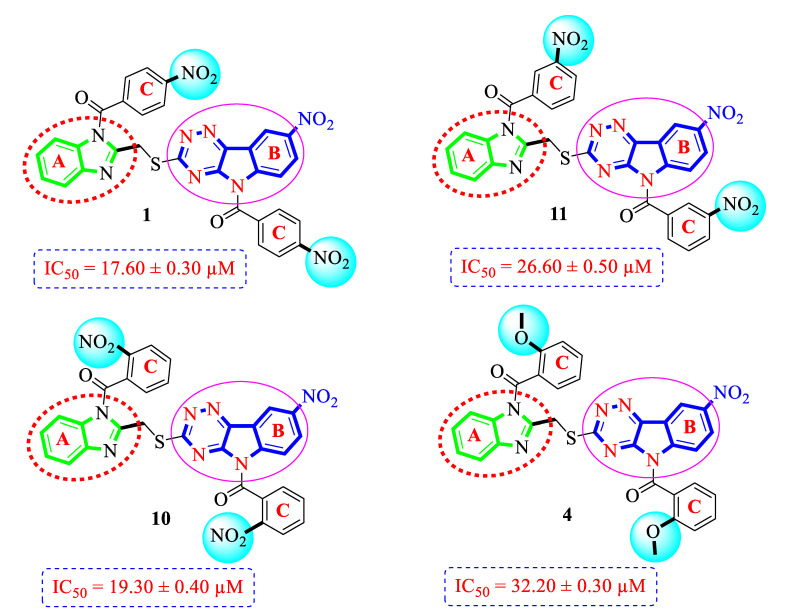
Structure-activity relationship (SAR) study of analogs **1**, **4**, **10**, and **11**.

Better inhibitory potentials were identified for analogs **1**, **10**, and **11**, with nitro groups on the different locations of both aryl parts C. Among these nitro-substituted analogs, the analog **1** bearing –NO_2_ group on the *para*-position of both aryl parts C (one aryl part is linked to benzimidazole nitrogen, while the other aryl moiety is bonded to the nitrogen of triazinoindole) was found to be an active inhibitor of the urease enzyme. However, analog **10**, bearing, at the –NO_2_ moiety, the *ortho*-position of both aryl parts C, showed approximately comparable potency with respect to analog **1**, but showed enhanced inhibitory potential compared to analog **11**, which has the –NO_2_ group at the *meta*-position of both aryl parts C. This small difference in potency of these three analogs is due to different positions of the –NO_2_ group around the aryl **C** parts. The decrease in activity of analog **10** was observed by replacing the *meta*-nitro group with the methoxy group, as in the case of analog **4**. The enhanced activity of analog **10** might be due to the EW nature of the –NO_2_ group, which helps in yielding a better interaction of analog **10** within the enzyme’s pocket (Figure 2).

**Figure 3:**
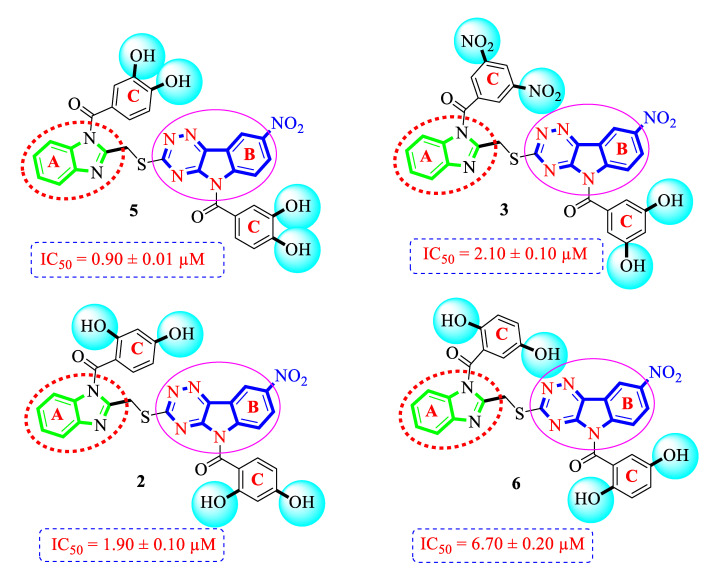
Structure-activity relationship (SAR) of di-hydroxyl groups bearing analogs **2**, **3**, **5** and **6**.

Analogs (**2**, **3**, **5**, and **6**) being substituted with di-hydroxy groups at various positions of both aryl parts C exhibited varying degrees of inhibitory potentials. The analog **5** that has di-hydroxy substitutions at the 3,4-position of both aryl parts C was identified as the potent inhibitor of the urease enzyme among di-hydroxy groups bearing analogs. The di-hydroxy substitutions at the 3,4-position of both aryl portions C of analog **5** are found to be more favorable, making them capable of forming a number of significant interactions with the active site of enzymes. However, analog **2** bearing di-hydroxy substitutions at the 2,4-position of both aryl part Cs showed almost one-fold less activity as compared to analog **5**. The activity of analog **5** was further decreased by shifting the *para*-hydroxyl group to the second *meta*-position of aryl part Cs as in the case of analog **3**, which holds di-hydroxy substitutions at the 3,5-position of both aryl parts C. Among di-hydroxy substitutions bearing analog, the analog 6 bearing di-hydroxy substitutions at the 2,5-position of both aryl parts C was shown as the least inhibitor of the urease enzyme. This discrepancy in the inhibitory potential of di-hydroxy groups bearing analogs might be due to the different positions of di-hydroxy groups around both aryl parts C (Figure 3).

**Figure 4:**
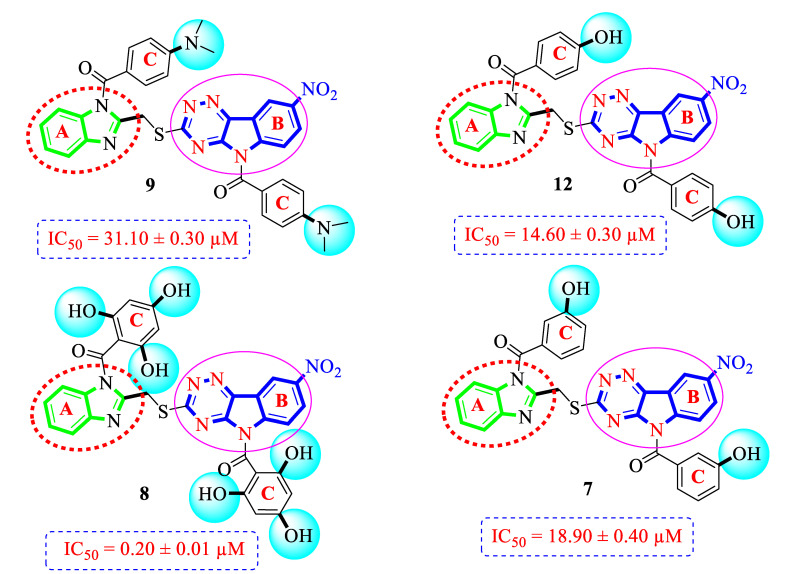
SAR study of analogs **7**, **8**, **9**, and **12**.

Analog **8**, bearing tri-hydroxy substitutions at the 2,4,6-position of both aryl parts C emerged as the most active competitor of the urease enzyme and displayed twenty-fold more potency than the standard thiourea drug. The urease inhibitory potentials of analog **8** declined by the de-attachment of two hydroxyl groups present at the 2,6-position of both aryl parts C, as in the case of analog **12** (which holds only one hydroxyl group at the 4-position of both aryl parts C). Furthermore, it was also observed that the replacement of a *para*-hydroxyl group of both aryl parts C of analog **12;** the di-methyl amino group, as in analog **9**, also resulted in decreased activity. This indicates that the dimethyl amino group is not actively contributing to the activity. By comparing the analog **7** bearing meta-hydroxyl group on both aryl parts C with its structurally similar analog **12**, which holds the hydroxyl group at the para-position of both aryl parts C, analog **12** showed enhanced activity compared to its counterpart, analog **7**. This difference in potency might be due to the different positions of the hydroxyl group around both aryl parts C (Figure 4).

#### 2.2.3. Category “B”

**Figure 5:**
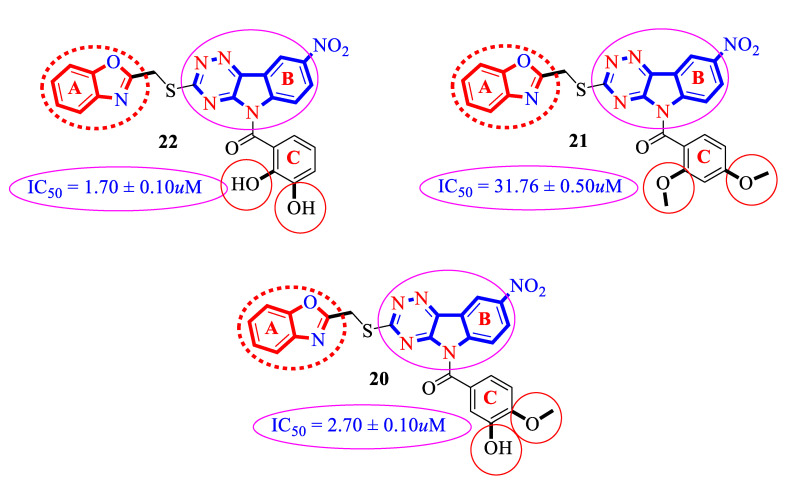
Structure-activity relationship (SAR) study of analogs **20**, **21**, and **22**.

Analogs (**13**–**25**) belong to category “B”. In category “B”, analog **22**, having di-hydroxy substitutions at the 2,3-position of the aryl ring, showed a many times enhanced inhibitory potential, compared to the standard thiourea drug. The inhibitory potential of analog **22** was dropped down by the removal of the *ortho*-hydroxyl group, followed by the incorporation of the methoxy group at the *para*-position of aryl ring C, as in the case of analog **20**. However, the incorporation of two methoxy groups at the 2,4-position of aryl ring C of analog **21** resulted in decreased activity when compared to other hydroxy groups bearing analogs **20** and **22**. This lower potency of analog **21** in comparison to analogs **20** and **22** might be due to steric hindrance, caused by the bulky methoxy groups and, hence, resulting in decreased potency (Figure 5).

**Figure 6:**
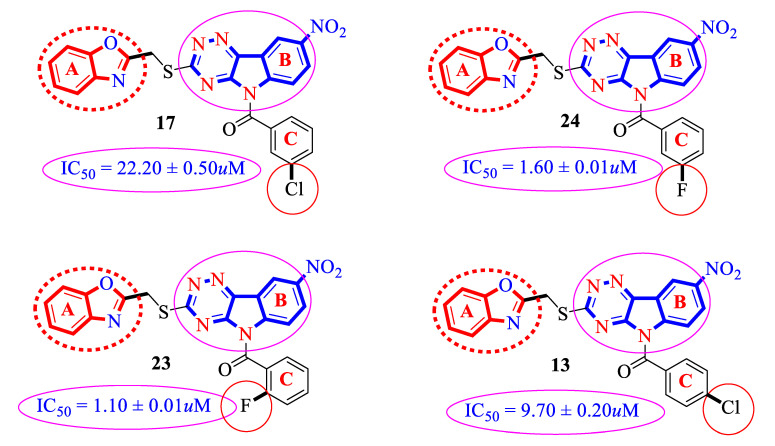
Structure-activity relationship study of halogen-substituted analogs **13**, **17**, **23**, and **24**.

The urease inhibitory activity of the halogen-substituted analogs with fluoro moiety was superior to that of the analogs with the chloro moiety. Compound **23** bearing ortho-fluoro substitution at aryl ring exhibited nine-fold more potency than standard thiourea drug. Although structurally identical, analog **24** with a meta-fluoro substitution at the aryl ring demonstrated decreased activity, suggesting that the substitution of a fluoro group at the aryl ring’s para-position may facilitate better contact with the active sites of enzymes. By comparing analogs **17** (bearing *meta*-chloro substitution) and **24** (bearing *meta*-fluoro substitution), the analog 24-bearing meta-fluoro substitution showed superior activity to analog 17. This difference might be due to the different nature (-F is a stronger EWG than –Cl) of the substituent around the aryl ring. On the other hand, the *para*-chloro-substituted analog **13** showed better activity than its structurally similar counterpart **17**, indicating that different positions of the same substituent around aryl ring C also resulted in varied potency (Figure 6).

**Figure 7:**
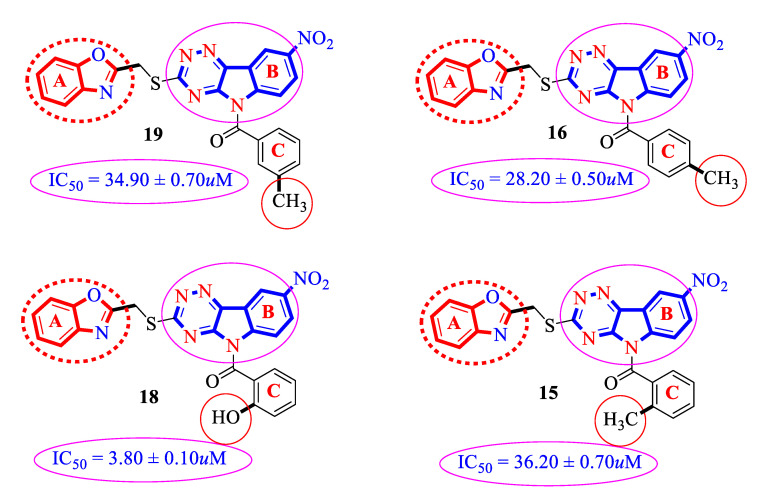
SAR study for analogs **15**, **16**, **18**, and **19**.

The analog **18** bearings of *ortho*-hydroxy substitution at the aryl ring exhibited an inhibitory potential many times more than the standard thiourea drug. However, the inhibitory potentials of analog **18** sharply decreased by replacing the *ortho*-hydroxy group with the methyl group, as in the case of compound **15**, showing that the methyl group is not actively contributing to this activity. Moreover, the inhibitory potential was increased by shifting the methyl group from its *ortho*-position to the *meta*-position of the aryl ring, as in compound **19**. In analog **16**, the methyl group was added to the para-position of the aryl ring, which further boosted the activity. The difference in inhibitory potential found in these three analogs might be due to the different positions of the methyl group around the aryl ring (Figure 7).

On the basis of the aforementioned observation that the inhibitory potential possessed by newly synthesized triazinoindole-based benzimidazole/benzoxazole analogs was greatly influenced by changing the nature, electron-donating/electron-withdrawing effect, number/s, and position of the substituents around the aryl part C. Moreover, it was also noteworthy that analogs bearing hydroxyl groups (present at different positions on the aromatic ring, such as the *para/ortho* and *meta*-positions) as well as the fluoro group at the appropriate position of the aryl part C seemed to be the better competitor of urease enzyme and was shown to be many times more potent than standard thiourea drugs.

### 2.3. Docking Study

**Figure 8:**
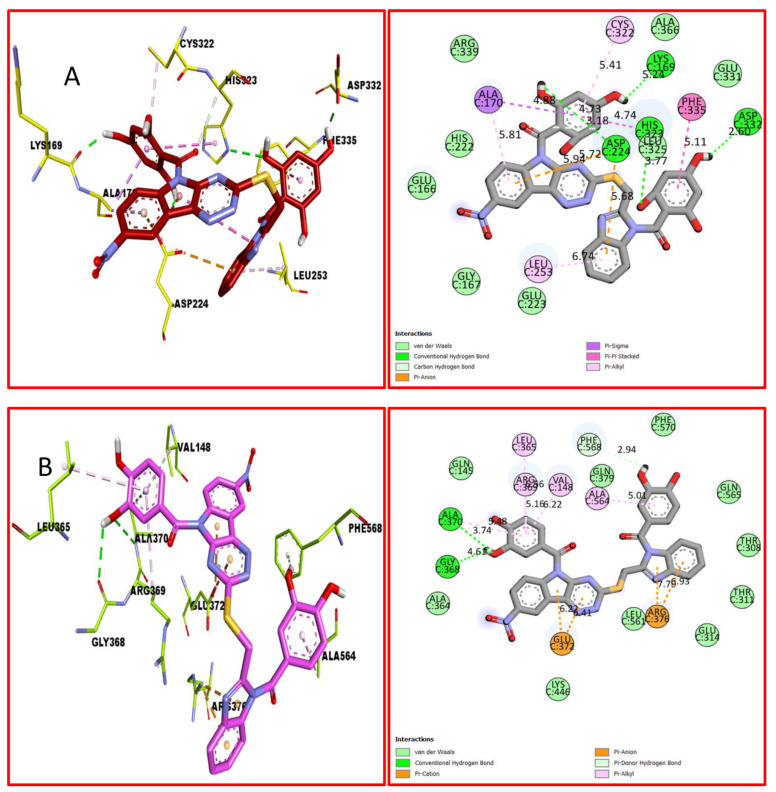
Protein-ligand interactions (PLI) profile most active analogs of triazinoindole-based benzimidazole category. (**A**) for compound **8**, while (**B**) for compound **5** against urease enzyme.

Molecular docking was used to investigate the active component in newly afforded triazinoindole-based benzimidazole/benzoxazole analogs against the urease enzyme. When evaluated, newly afforded analogs with either a tri-hydroxy substitution or a fluoro moiety displayed improved inhibitory potentials (in vitro). These tri-hydroxy and fluoro-substituted analogs were subsequently subjected to a molecular docking study to investigate their binding site, and the results show that the most potent analogs **8**, **5**, **22**, and **23** not only display better potency (in vitro) but also established numerous significant interactions (in silico) with the active site of amino acid. According to the PLI profile of the most active analog **8** bearing tri-hydroxy substitutions at the 2,4,6-position of both aryl rings C, analog **8** displays several significant interactions with urease enzymes including Asp332, Phe335, Leu253, Asp224, Ala170, Lys169 and Cys322 as shown in (Figure 8A), while analog **5** against urease enzyme adopted several key interactions including Phe568, Ala564, Arg378, Glu372, Arg359, Gly368, Ala370, Leu365 and Val148 as shown in (Figure 8B).

**Figure 9:**
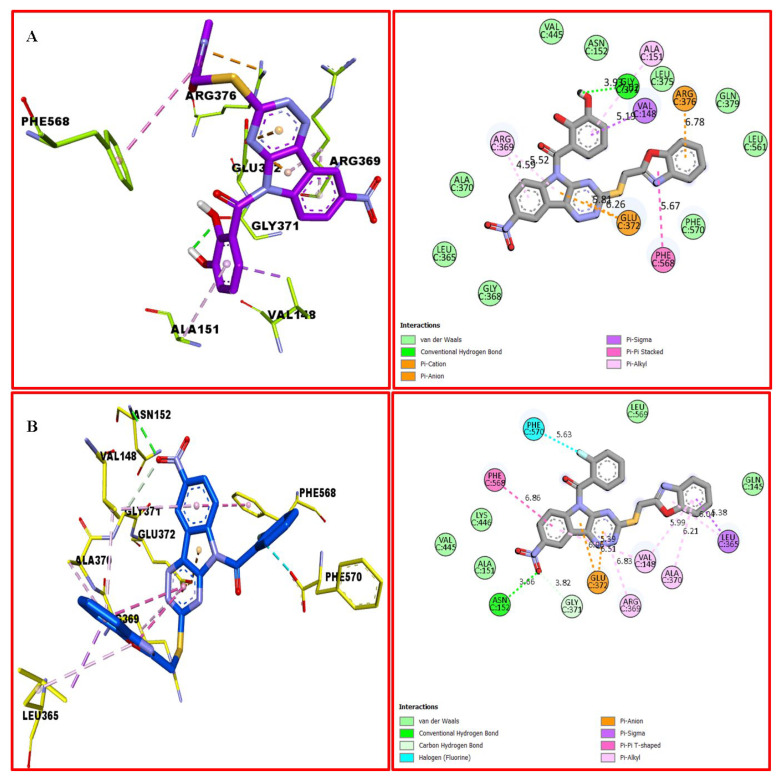
Protein-ligand interactions (PLI) profile of the most active analogs of the triazinoindole-based benzoxazole category. (**A**) shows compound **22**, while (**B**) shows compound **23** against the urease enzyme.

The third most active analog **22**, which has tri-hydroxy substitutions at the positions 2,4,6 of aryl ring C, shows a variety of noteworthy interactions with the urease enzyme including Phe568, Arg378, Glu372, Arg369, Gly371, Val148, and Ala151 as shown in (Figure 9A), while analog **23**, against the urease enzyme, adopted several key interactions such as Asn152, Ala148, Gly371, Glu372, Ala370, Leu2365, Arg369, Phe570, and Phe568 as shown in (Figure 9B).

**Figure 10:**
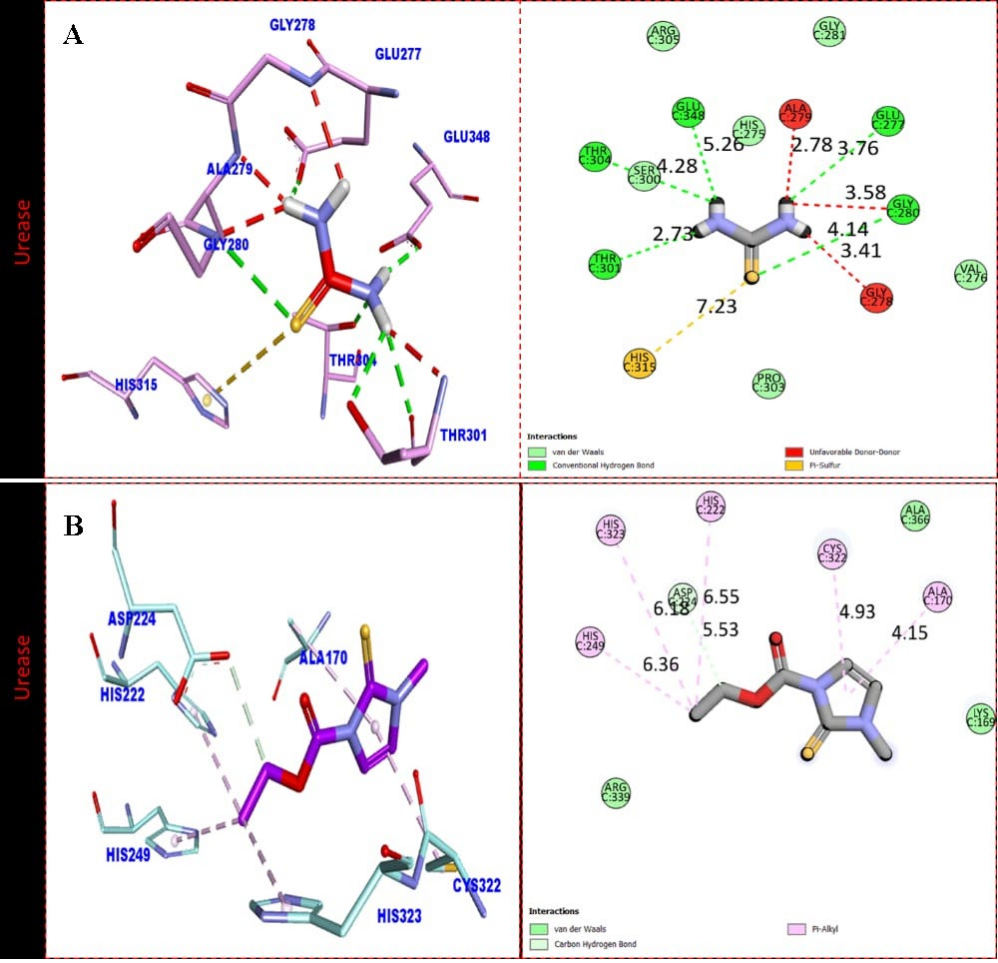
Protein-ligand interaction (PLI) profile of standard thiourea and its derivative. (**A**) is for standard thiourea, while (**B**) is the thiourea drug against the urease enzyme.

In the case of the standard thiourea drug (Figure 10A) and its derivative (Figure 10B), when subjected to molecular docking, these established important interactions with the catalytic residue of the amino acid of the urease enzyme. Thiourea interacts with the catalytic residue of amino acid through hydrogen bonding and forms five hydrogen bonds with the catalytic residue of amino acid. Similarly, a thiourea-based drug (Figure 10B) established key interactions with the active site of the urease enzyme, including the carbon hydrogen bond, pi-alkyl, and van der Waals interactions. By comparing the interactions developed by the active analogs of the series, such as **8**, **5**, **22**, and **23**, with thiourea and its derivative, the potent analogs **8**, **5**, **22**, and **23** established some additional interactions (pi-cation, pi-anion, pi-sigma, pi-pi stacking, pi-sulfur, and halogen bond interaction) with the active residue of amino acid of the urease enzyme; therefore, these analogs were found to be many times more potent than the standard thiourea drug.

## 3. Experimental

### 3.1. General Information

Every chemical was obtained from genuine suppliers (Merck, Sigma) and was used without any refinement. A clear and authentic TLC was used, whereas the Buchi M-560 was employed to determine the melting point. Additionally, the ^1^H-NMR and ^13^C-NMR spectra of synthetically produced derivatives were captured using the Bruker 500 Ultra-shield plus NMR (500 MHz). The chemical shift values for TMS were expressed in ppm and the coupling constant was expressed in Hz. A high-resolution electron impact mass spectrophotometer was used to measure the mass of the synthesized derivatives.

### 3.2. General Procedure for the Formation of Triazinoindole-Based Benzimidazole/Benzoxazole Derivatives *(**1**–**25**)*

The synthesis of triazinoindole-based benzimidazole/benzoxazole derivatives (**1**–**25**) was completed in several steps. In the first step, 3-mercapto triazinoindole intermediate (**III**) was afforded by the reaction of isatin (**I**) (1 equivalent) with thiosemicarbazide (**II**) (1 equivalent) in MeOH (10 mL), under the catalytic amount of potassium carbonate (0.6 mmol). On the other hand, 1,2-diamine benzene (IVA)/2-amino phenol (VIA) was cyclized in 4N HCl solution (10 mL) on stirring at the refluxing temperature over a pre-heated sand bath, to access the formation of the 2-(chloromethyl)-1H-benzo[d]imidazole (**IVB**)/benzoxazole based intermediate (**VIB**). In the next step, the 3-mercapto triazinoindole intermediate (**III**) (1 equivalent) was directly subjected to dehydrogenation upon the sequential addition of 2-(chloromethyl)-1H-benzo[d]imidazole (**IVB**) (1 equivalent) and triethylamine (few drops) in ethanol (10 mL) to afford a synthesis of triazinoindole bearing benzimidazole intermediate (**V**). Furthermore, an intermediate (**III**) (1 equivalent) was also treated with a benzoxazole-based intermediate (**VIB**) (1 equivalent) in ethanol (10 mL), followed by the addition of the catalytic amount of triethylamine (a few drops) to yield the triazinoindole-based benzoxazole intermediate (**VII**). In the final step, triazinoindole-based benzimidazole (**V**) (1 equivalent) and triazinoindole-based benzoxazole (**VII**) (1 equivalent) with different substituted benzoyl chloride (2 equivalent with intermediate (**V**), adding 1 equivalent with the intermediate (**VII**) in ethanol (10 mL) under the catalytic action of triethylamine (a few drops). We were pleased to see that stirring the resultant residue at the refluxing temperature provided the necessary yield for the synthesis of the required triazinoindole bearing benzimidazole (**1**–**12**) and benzoxazole hybrid scaffolds (**13**–**25**).

### 3.3. Urease Inhibition Assay

First, 5 μL of the test compounds were added to the reaction mixtures, which also contained 25 μL of enzyme solution and 55 μL of buffer containing 100 mM urea, then the mixtures were incubated in 96-well plates at 30 °C for 15 min (0.5 mM concentration). For the kinetics analysis, urea concentrations were changed from 2 to 24 mM. Urease activity was determined by measuring ammonia generation using Weatherburn’s indophenol method. In a nutshell, each well received 45 μL of the 0.005% *w/v* sodium nitroprusside, phenol reagent 1% *w/v* phenol, and 70 μL of the alkali reagent—0.1% active chloride NaOCl and 0.5% *w/v* NaOH. Using a microplate reader, rising absorbance at 630 nm was observed after 50 min (Molecular Devices, San Jose, CA, USA). In a final volume of 200 μL, each reaction was carried out three times. Using the software SoftMaxPro, the results (change in absorbance per minute) were processed (Molecular Devices, San Jose, CA, USA). The experiments were run at pH 6.8 throughout. The formula 100-(ODtest well/ODcontrol) 100 was used to calculate the percentage of inhibition. The preferred standard inhibitor was thiourea [49].

### 3.4. Assay Protocol for Docking Study

Utilizing Discovery Studio Visualizer’s (DSV) MGL tool 1.5.7 and AutoDock Vina, a molecular docking study was carried out. In this work, the synthetic analogs were tested against the urease enzyme. These enzymes’ structures were found in the Protein Data Bank (PDB) by using the search code **4ubp**. The target protein and the prepared ligand were both saved in PDB format after the first step of protein preparation, which involved utilizing DSV to remove the water molecules and existing ligands. The process was then continued in AutoDock, where the protein was charged with polar hydrogen, Kollman, and Gasteiger charges. Additionally, the chosen ligand was constructed using a torsion tree to find the root. Finally, a configuration file was created and saved in the same docking folder along with the X, Y, and Z axis for both the ligand and protein in PDBQT format. The ligand was then generated in various postures using a command line, yielding a total of 9 distinct poses in the PDBQT format. The dock protein and ligand were then opened in DSV to determine the ligand-enzyme active site interaction. The given article summarizes the additional information [50]. Details about thiourea drugs [51]; Spectral analysis are provided in the supplementary materials.

## 4. Conclusions

In conclusion, we have created a straightforward method for producing triazinoindole, which includes benzimidazole/benzoxazole, by simply condensing 3-mercapto triazinoindole with a substrate of benzimidazole/benzoxazole. The scope and diversity of this practical synthetics approach work well with the variety of benzoyl chloride. It is a viable alternative for the synthesis of triazinoindole-bearing benzimidazole/benzoxazole analogs, due to its characteristics of quick reaction time, simple operation, mild reaction conditions, and high efficiency. Additionally, hybrid analogs (**1**–**25**) of triazinoindole containing benzimidazole/benzoxazole were tested in vitro for their ability to inhibit urease, according to a technique that has been documented in the literature. Among the series, compounds **8** (bearing tri-hydroxy substitutions on both aryl parts C), **5** (bearing di-hydroxy substitutions at the 3,4-position of both aryl parts C), **22** (di-hydroxy substitutions at the 2,3-position of aryl part C) and **23** (bearing *ortho*-fluoro substitution at aryl part C) with IC_50_ values of 0.20 ± 0.01, 0.90 ± 0.01, 1.70 ± 0.10, and 1.10 ± 0.01 μM were found to be many times more potent than thiourea as a standard inhibitor (IC_50_ = 21.86 ± 0.40 µM). The enhanced inhibitory profile of these scaffolds may be caused by the presence of the tri-, di-hydroxy-, and fluoro groups at various positions on the phenyl ring C. By involving the oxygen of the tri-, di-hydroxy group, and fluoro group in hydrogen bonding with the active sites of enzymes, these scaffolds have demonstrated a much better inhibitory profile. Molecular docking research was employed to identify the binding interactions between the most active scaffolds and the enzyme active site.

## Figures and Tables

**Table 1:** Triazinoindole-based benzimidazole/benzoxazole analogs, with different substituent(s) and IC_50_ values, against the urease enzyme.

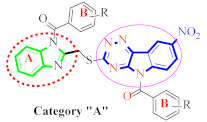					
**S.NO**	**R**	**IC_50_ ± SEM** **(μM)**	**S.NO**	**R**	**IC_50_ ± SE** **(μM)**
**1**	4-nitro	17.60 ± 0.30	**2**	2,4-dihydroxy	1.90 ± 0.10
**3**	3,5-dihydroxy	2.10 ± 0.10	**4**	2-methoxy	32.20 ± 0.30
**5**	3,4-dihydroxy	0.90 ± 0.01	**6**	2,5-dihydroxy	6.70 ± 0.20
**7**	3-hydroxy	18.90 ± 0.40	**8**	2,4,6-trihydroxy	0.20 ± 0.01
**9**	4-dimethylamino	31.10 ± 0.30	**10**	2-nitro	19.30 ± 0.40
**11**	3-nitro	26.60 ± 0.50	**12**	4-hydroxy	14.60 ± 0.30
**Standard Thiourea**					**21.86 ± 0.40 μM**
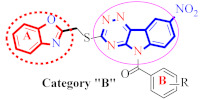					
**13**	4-chloro	9.70 ± 0.20	**14**	4-methyl ethanoate	23.90 ± 0.50
**15**	2-methyl	36.20 ± 0.70	**16**	4-methyl	28.20 ± 0.50
**17**	3-chloro	22.20 ± 0.50	**18**	2-hydroxy	3.80 ± 0.10
**19**	3-methyl	34.90 ± 0.70	**20**	3-hydroxy-4-methoxy	2.70 ± 0.10
**21**	2,4-dimethoxy	31.76 ± 0.50	**22**	2,3-dihydroxy	1.70 ± 0.10
**23**	2-fluoro	1.10 ± 0.01	**24**	3-fluoro	1.60 ± 0.01
**25**	3-nitro	21.20 ± 0.40	**-**	-	-
**Standard Thiourea**					**21.86 ± 0.40 μM**

## Data Availability

The data will be available on request.

## Supplementary Materials

The following supporting information can be downloaded at: https://www.mdpi.com/article/10.3390/molecules27196580/s1, detail about thiourea based drugs and spectral analysis of the compounds.

## Sample Availability

Not available.
