# Supplementary material for: New Triazinoindole Bearing Benzimidazole/Benzoxazole Hybrids Analogs as Potent Inhibitors of Urease: Synthesis, In Vitro Analysis and Molecular Docking Studies
Source: Molecules. 2022 Oct 4;27(19):6580. doi: 10.3390/molecules27196580 (PMC9571547; doi:10.3390/molecules27196580)
Supplement: Supplementary file 1 [file molecules-27-06580-s001.zip › molecules-1937311-supplementary.pdf]

Supplementary Materials

New Triazinoindole Bearing Benzimidazole/Benzoxazole Hybrids Analogs as Potent Inhibitors of Urease: Synthesis, In Vitro Analysis and Molecular Docking Studies

Detail about Thiourea Based Drugs

Thiourea is the classic bioisostere of urea functionality, in which oxygen is replaced by sulfur atom and represents one of the most important core structures incorporated into medicinal chemistry. This scaffold serves as an excellent building block in the discovery of new drug candidates with diverse therapeutic applications. There are many commercial examples (Figure S1) that carry thiourea group integrated into their structures such as thiocarlide, noxytiolin (antibacterial), thioacetazone (antitubercular), and thiopental (general anesthetic) [49].

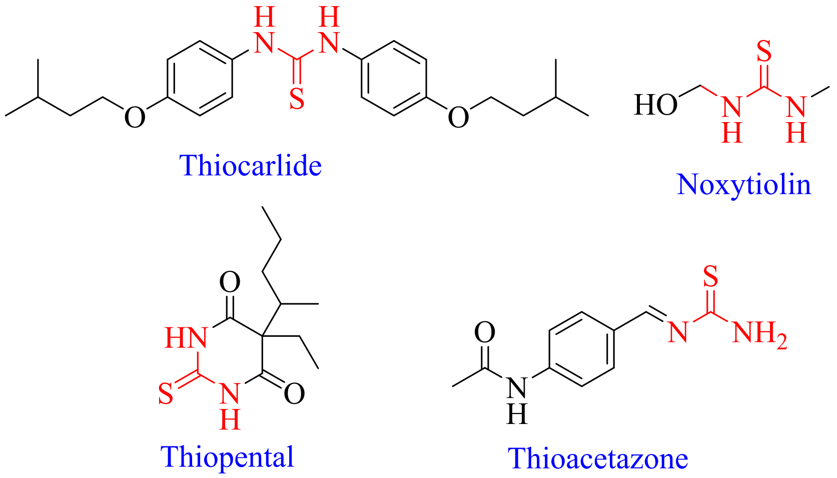

Figure S1. Examples for drugs carrying the thiourea moiety

Spectral Analysis

(8-nitro-3-(((1-(4-nitrobenzoyl)-1H-benzo[d]imidazol-2-yl)methyl)thio)-5H-[1,2,4]triazino[5,6-b]indol-5-yl)(4-nitrophenyl)methanone (1)

^{1}H NMR (500 MHz, DMSO-d_6): δ8.80(s, 1H, Triazinoindole-H),8.51 (d, *J*= 7.6Hz, 2H, Ar-H), 8.49 (d, *J* = 7.2Hz, 2H, Ar-H), 8.45 (d, *J* = 6.8Hz, 2H, Ar-H), 8.40 (d, *J* = 7.8Hz, 2H, Ar-H), 8.10 (d, *J* = 8.0Hz, 1H, Triazinoindole-H), 7.99 (d, *J*= 6.9Hz, 1H, Triazinoindole-H), 7.60 (dd, *J*= 6.6, 2.0Hz, 1H, Benzimidazole-H), 7.45 (dd, *J*= 7.6, 1.9Hz, 1H,

Benzimidazole-H), 7.39-7.32 (m, 1H, Benzimidazole-H), 7.17-7.11 (m, 1H, Benzimidazole-H), 4.38 (s, 2H, -HC-S).;^{13}C-NMR (125 MHz, DMSO-d6): δ170.3, 167.5, 167.3, 153.5, 153.3, 145.0, 141.7, 141.4, 139.0, 138.9, 138.5, 132.0, 130.6, 130.6, 130.4, 130.2, 126.5, 124.0, 124.0, 123.8, 123.8, 123.4, 123.4, 122.8, 122.8, 115.0, 113.9, 113.3, 111.7, 101.5, 32.6. HREI-MS: m/z [M]$^+$ calcd for $C_{31}H_{17}N_9O_8S$675.0740, found 675.0530.

(3-(((1-(2,4-dihydroxybenzoyl)-1H-benzo[d]imidazol-2-yl)methyl)thio)-8-nitro-5H-[1,2,4]triazino[5,6-b]indol-5-yl)(2,4-dihydroxyphenyl)methanone (2)

^{1} H NMR (500 MHz, DMSO-d_6): δ 9.43 (s, 1H, OH),9.40 (s, 1H, OH), 9.33 (s, 1H, OH), 9.30 (s, 1H, OH), 8.83 (s, 1H, Triazinoindole-H), 8.60 (d, *J*= 7.1 Hz, 1H, Ar-H), 8.58 (s, 1H, Ar-H), 8.54 (d, *J* = 7.0 Hz, 1H, Ar-H), 8.49 (d, *J* = 7.8 Hz, 1H, Ar-H), 8.43 (s, 1H, Ar-H), 8.40 (d, *J* = 6.8 Hz, 1H, Ar-H), 8.15 (d, *J* = 8.3 Hz, 1H, Triazinoindole-H), 8.03 (d, *J*= 7.9 Hz, 1H, Triazinoindole-H), 7.75 (d, *J*= 7.6 Hz, 2H, Benzimidazole-H), 7.56 (d, *J*= 7.2 Hz, 2H, Benzimidazole-H), 4.41 (s, 2H, -HC-S).; ^{13}C-NMR (125 MHz, DMSO-d6): δ171.3, 168.5, 168.3, 154.5, 154.3, 146.0, 142.7, 142.4, 140.0, 139.9, 139.5, 133.0, 132.6, 131.6, 130.9, 130.3, 127.5, 125.0, 124.0, 123.6, 123.6, 123.7, 123.2, 122.2, 122.0, 116.0, 114.9, 114.3, 112.7, 102.5, 41.6. HREI-MS: m/z [M]$^+$calcd for $C_{31}H_{19}N_7O_8S$ 649.1240, found 649.1158.

(3-(((1-(3,5-dihydroxybenzoyl)-1H-benzo[d]imidazol-2-yl)methyl)thio)-8-nitro-5H-[1,2,4]triazino[5,6-b]indol-5-yl)(3,5-dihydroxyphenyl)methanone (3)

^{1} H NMR (500 MHz, DMSO-d_6):): δ 9.41 (s, 1H, OH), 9.38 (s, 1H, OH), 9.30 (s, 1H, OH), 9.28 (s, 1H, OH), 8.82 (s, 1H, Triazinoindole-H), 8.59 (s, 1H, Ar-H), 8.56 (s, 1H, Ar-H), 8.52 (s, 1H, Ar-H), 8.47 (s, 1H, Ar-H), 8.39 (s, 1H, Ar-H),8.37 (s, 1H, Ar-H), 8.12 (d, *J* = 7.3 Hz, 1H, Triazinoindole-H), 8.00 (d, *J*= 7.5Hz, 1H, Triazinoindole-H), 7.73 (d, *J*= 8.6 Hz, 2H, Benzimidazole-H), 7.50 (d, *J*= 7.3 Hz, 2H, Benzimidazole-H), 4.35 (s, 2H, -HC-S).; ^{13}C-NMR (125 MHz, DMSO-d6): δ171.2, 168.4, 168.1, 154.4, 154.2, 146.3, 142.6, 142.3, 141.0, 139.7, 139.4, 133.2, 132.0, 131.3, 130.6, 130.1, 127.4, 125.2, 124.6, 123.5, 123.5, 123.5, 123.0, 122.1, 122.4, 116.3, 114.8, 114.2, 112.6, 102.4, 42.3. HREI-MS: m/z [M]$^+$calcd for $C_{31}H_{19}N_7O_8S$ 675.0740, found 675.0530.

(3-(((1-(2-methoxybenzoyl)-1H-benzo[d]imidazol-2-yl)methyl)thio)-8-nitro-5H-[1,2,4]triazino[5,6-b]indol-5-yl)(2-methoxyphenyl)methanone (4)

^{1} H NMR (500 MHz, DMSO-d_6): δ 8.70 (s, 1H, Triazinoindole-H), 8.06 (d, *J* = 7.3 Hz, 1H, Triazinoindole-H), 8.02 (d, *J*= 6.9 Hz, 1H, Triazinoindole-H), 7.60 (dd, *J*= 7.1, 1.8 Hz, 1H, Ar-H), 7.56-7.52 (m, 1H, Ar-H), 7.50 (dd, *J* = 7.7, 2.1 Hz, 1H, Ar-H), 7.48-7.44 (m, 1H, Ar-H), 7.41 (dd, *J* = 7.9, 1.8 Hz, 1H, Ar-H), 7.39-7.35 (m, 1H, Ar-H),7.33 (dd, *J* = 6.8, 2.0Hz, 1H, Ar-H), 7.30-7.29 (m, 1H, Ar-H), 7.26 (d, *J*= 8.6 Hz, 2H, Benzimidazole-H), 7.22 (d, *J*= 7.4 Hz, 2H, Benzimidazole-H), 4.30 (s, 2H, -HC-S), 3.30 (s, 3H, -OCH3), 3.25 (s, 3H, -OCH3).; ^{13}C-NMR (125 MHz, DMSO-d6): 170.2, 168.2, 168.6, 154.7, 154.3, 145.3, 143.6, 142.5, 141.8, 140.7, 139.2, 134.2, 133.0, 132.3, 131.6, 131.1, 128.4, 126.2, 125.6, 124.5, 124.5, 123.3, 123.1, 122.5, 122.3, 117.3, 115.8, 115.2, 113.6, 103.4, 55.0, 54.7, 33.8. HREI-MS: m/z [M]$^+$calcd for $C_{33}H_{23}N_7O_6S$645.1930, found 645.1870.

(E)-N'-(3,4-dihydroxybenzylidene)benzofuran-3-carbohydrazide (5)

^{1}H NMR (500 MHz, DMSO-d_6):): δ 9.42 (s, 1H, OH),9.39 (s, 1H, OH), 9.31 (s, 1H, OH), 9.28 (s, 1H, OH), 8.82 (s, 1H, Triazinoindole-H), 8.61 (d, *J*= 7.4Hz, 1H, Ar-H), 8.57 (s, 1H, Ar-H), 8.52 (d, *J* = 7.7 Hz, 1H, Ar-H), 8.46 (d, *J* = 7.9 Hz, 1H, Ar-H), 8.41 (s, 1H, Ar-H), 8.37 (d, *J* = 7.8 Hz, 1H, Ar-H), 8.13 (d, *J* = 8.3 Hz, 1H, Triazinoindole-H), 8.00 (d, *J*= 8.9 Hz, 1H, Triazinoindole-H), 7.70 (d, *J*= 8.6 Hz, 2H, Benzimidazole-H), 7.47 (d, *J*= 7.3 Hz, 2H, Benzimidazole-H), 4.36 (s, 2H, -HC-S).; ^{13}C-NMR (125 MHz, DMSO-d6): δ 171.2, 168.3, 168.0, 154.4, 154.2, 146.1, 142.6, 142.3, 140.2, 139.8, 139.4, 133.7, 132.5, 131.5, 130.8, 130.2, 127.4, 125.1, 124.6, 123.5, 123.4, 123.3, 123.1, 122.0, 121.0, 116.7, 114.8, 114.2, 112.6, 102.4, 40.5. HREI-MS: m/z [M]$^+$calcd for $C_{31}H_{19}N_7O_8S$649.4478, found 649.4390.

(3-(((1-(2,5-dihydroxybenzoyl)-1H-benzo[d]imidazol-2-yl)methyl)thio)-8-nitro-5H-[1,2,4]triazino[5,6-b]indol-5-yl)(2,5-dihydroxyphenyl)methanone (6)

^{1}H NMR (500 MHz, DMSO-d_6): δ 9.40 (s, 1H, OH),9.37 (s, 1H, OH), 9.29 (s, 1H, OH), 9.25 (s, 1H, OH), 8.81 (s, 1H, Triazinoindole-H), 8.57 (d, *J*= 7.2 Hz, 1H, Ar-H), 8.53 (s, 1H, Ar-H), 8.50 (d, *J* = 6.7 Hz, 1H, Ar-H), 8.43 (d, *J* = 7.5 Hz, 1H, Ar-H), 8.41 (s, 1H, Ar-H), 8.33 (d, *J* = 8.8 Hz, 1H, Ar-H), 8.13 (d, *J* = 8.3 Hz, 1H, Triazinoindole-H), 8.00 (d, *J*= 8.9 Hz, 1H, Triazinoindole-H), 7.73 (d, *J*= 8.3 Hz, 2H, Benzimidazole-H), 7.52 (d, *J*= 7.4 Hz, 2H, Benzimidazole-H), 4.26 (s, 2H, -HC-S).; ^{13}C-NMR (125 MHz, DMSO-d6): δ 171.4, 168.6, 168.7, 154.3, 154.1, 146.0, 142.5, 142.2, 140.1, 139.7, 139.3, 133.6, 132.4, 131.4, 130.7, 130.9, 127.8, 125.0, 124.5, 123.4, 123.3, 123.2, 123.0, 122.4, 121.9, 116.6, 114.7, 114.1, 112.5, 102.3, 33.4. HREI-MS: m/z [M]$^+$calcd for $C_{31}H_{19}N_7O_8S$ 649.0970, found 649.0820.

(3-(((1-(2-bromo-4-nitrobenzoyl)-1H-benzo[d]imidazol-2-yl)methyl)thio)-8-nitro-5H-[1,2,4]triazino[5,6-b]indol-5-yl)(2-bromo-4-nitrophenyl)methanone (7)

^{1}H NMR (500 MHz, DMSO-d_6): δ 8.70 (dd, *J* = 6.8, 1.9Hz, 1H, Ar-H), 8.56 (d, *J*= 1.9 Hz, 1H, Ar-H), 8.15 (dd, *J* = 7.2, 1.9 Hz, 1H, Triazinoindole-H), 8.05 (d, *J* = 7.3 Hz, 1H, Ar-H), 7.98 (s, 1H, Triazinoindole-H), 7.88 (d, *J* = 7.0 Hz, 1H, Triazinoindole-H), 7.41 (d, *J* = 1.7 Hz, 1H, Ar-H), 7.28 (d, *J* = 6.8, 1.7 Hz, 1H, Ar-H), 7.02 (d, *J*= 7.1 Hz, 1H, Ar-H), 6.98 (d, *J*= 7.1 Hz, 2H, Benzimidazole-H), 6.83 (d, *J*= 6.8 Hz, 2H, Benzimidazole-H), 4.72 (s, 2H, -HC-S).; ^{13}C-NMR (125 MHz, DMSO-d6): δ 169.6, 167.7, 157.3, 154.4, 148.4, 148.1, 147.5, 144.2, 141.9, 141.6, 141.2, 140.8, 130.9, 127.2, 127.1, 124.7, 123.0, 122.7, 122.2, 122.3, 119.5, 116.5, 116.4, 116.2, 116.0, 115.7, 113.5, 112.7, 109.5, 108.5, 44.7. HREI-MS: m/z [M]$^+$calcd for $C_{31}H_{15}Br_2N_9O_8S$ 617.2180, found 617.2378.

(8-nitro-3-(((1-(2,4,6-trihydroxybenzoyl)-1H-benzo[d]imidazol-2-yl)methyl)thio)-5H-[1,2,4]triazino[5,6-b]indol-5-yl)(2,4,6-trihydroxyphenyl)methanone (8)

^{1}H NMR (500 MHz, DMSO-d_6):): δ 9.49 (s, 1H, OH), 9.46 (s, 1H, OH), 9.38 (s, 1H, OH), 9.36 (s, 1H, OH), 9.33 (s, 1H, OH), 9.31 (s, 1H, OH), 8.87 (s, 1H, Triazinoindole-H), 8.63 (s, 1H, Ar-H), 8.61 (s, 1H, Ar-H), 8.51 (s, 1H, Ar-H), 8.47 (s, 1H, Ar-H), 8.18 (d, *J* = 7.3 Hz, 1H, Triazinoindole-H), 8.08 (d, *J*= 7.7 Hz, 1H, Triazinoindole-H), 7.65 (d, *J*= 6.4 Hz, 2H, Benzimidazole-H), 7.43 (d, *J*= 6.8 Hz, 2H, Benzimidazole-H), 4.50 (s, 2H, -HC-S).; ^{13}C-NMR (125

MHz, DMSO-d6): δ 172.3, 170.5, 169.3, 156.5, 155.3, 148.0, 144.7, 143.4, 142.0, 141.9, 140.5, 135.0, 134.6, 133.6, 132.9, 131.3, 129.5, 128.0, 126.0, 125.6, 125.6, 124.7, 124.2, 123.2, 123.0, 118.0, 117.9, 116.3, 114.7, 104.5, 34.6. HREI-MS: m/z $[M]^+$calcd for $C_{31}H_{19}N_7O_{10}S$ 681.7740, found 681.7630.

(3-(((1-(4-(dimethylamino)benzoyl)-1H-benzo[d]imidazol-2-yl)methyl)thio)-8-nitro-5H-[1,2,4]triazino[5,6-b]indol-5-yl)(4-(dimethylamino)phenyl)methanone (9)

^{1}H NMR (500 MHz, DMSO-d_6): δ 8.75 (s, 1H, Triazinoindole-H), 8.45 (d, *J*= 7.7Hz, 2H, Ar-H), 8.44 (d, *J* = 7.9 Hz, 2H, Ar-H), 8.39 (d, *J* = 7.8 Hz, 2H, Ar-H), 8.35 (d, *J* = 7.1 Hz, 2H, Ar-H), 8.01 (d, *J* = 7.0 Hz, 1H, Triazinoindole-H), 7.97 (d, *J*= 7.6 Hz, 1H, Triazinoindole-H), 7.55 (d, *J*= 6.9 Hz, 2H, Benzimidazole-H), 7.41 (d, *J*= 7.0 Hz, 2H, Benzimidazole-H), 4.23 (s, 2H, -HC-S), 2.19 (s, 6H, -NCH3),2.15 (s, 6H, -NCH3).; ^{13}C-NMR (125 MHz, DMSO-d6): δ 169.3, 166.5, 166.3, 152.5, 152.3, 144.0, 140.7, 140.4, 138.0, 137.9, 137.5, 131.0, 129.6, 129.6, 129.4, 129.2, 125.5, 123.0, 123.0, 122.8, 122.8, 122.4, 122.4, 121.8, 121.8, 114.0, 112.9, 112.3, 110.7, 100.5, 39.5, 39.1, 31.6. HREI-MS: m/z $[M]^+$calcd for $C_{35}H_{29}N_9O_4S$ 671.9974, found 671.9720.

(8-nitro-3-(((1-(2-nitrobenzoyl)-1H-benzo[d]imidazol-2-yl)methyl)thio)-5H-[1,2,4]triazino[5,6-b]indol-5-yl)(2-nitrophenyl)methanone (10)

^{1}H NMR (500 MHz, DMSO-d_6): δ 8.89 (dd, *J* = 7.3, 2.8 Hz, 1H, Ar-H), 8.67 (dd, *J*= 6.8, 1.9 Hz, 1H, Ar-H), 8.54 (dd, *J*= 6.7, 1.2 Hz, 1H, Ar-H), 8.53-8.52 (m, 1H, Ar-H), 8.21-8.18 (m, 1H, Ar-H), 8.12-8.09 (m, 1H, Ar-H), 8.03 (dd, *J*= 8.9, 2.4 Hz, 1H, Ar-H), 7.83 (s, 1H, Triazinoindole-H), 7.80 (d, *J*= 6.4 Hz, 1H, Triazinoindole-H), 7.73-7.70 (m, 1H, Ar-H), 7.95 (dd, *J* = 8.7, 1.4 Hz, 1H, triazinoindole-H), 7.07 (d, *J* = 7.2 Hz, 2H, Benzimidazole-H), 7.02 (d, *J*= 7.1 Hz, 2H, Benzimidazole-H), 4.38 (s, 2H, -HC-S).; ^{13}C-NMR (125 MHz, DMSO-d6): δ 168.4, 167.1, 148.7, 146.5, 142.1, 141.5, 137.6, 136.6, 136.4, 135.0, 134.9, 134.9, 134.4, 133.8, 133.6, 133.6, 132.9, 130.1, 129.6, 127.5, 126.9, 126.7, 125.9, 125.9, 120.6, 118.5, 118.1, 117.5, 116.0, 112.1, 40.0. HREI-MS: m/z $[M]^+$ calcd for $C_{31}H_{17}N_9O_8S$ 675.0740, found 675.0530.

(8-nitro-3-(((1-(3-nitrobenzoyl)-1H-benzo[d]imidazol-2-yl)methyl)thio)-5H-[1,2,4]triazino[5,6-b]indol-5-yl)(3-nitrophenyl)methanone (11)

^{1}H NMR (500 MHz, DMSO-d_6):), 8.71 (s, 1H, Triazinoindole-H), 8.51 (dd, *J*= 7.3, 1.7 Hz, 1H, Ar-H), 8.51 (t, *J* = 7.9 Hz, 1H, Ar-H), 8.45 (dd, *J* = 8.0, 2.4 Hz, 1H, Ar-H), 8.38 (dd, *J* = 7.0, 2.1 Hz, 1H, Ar-H), 8.35 (t, *J* = 8.3, 1.9 Hz, 1H, Ar-H), 8.23 (dd, *J* = 8.2, 2.3 Hz, 1H, Ar-H), 8.05 (d, *J* = 7.0 Hz, 1H, Triazinoindole-H), 8.01 (d, *J*= 7.7 Hz, 1H, Triazinoindole-H), 7.68 (d, *J*= 8.3 Hz, 2H, Benzimidazole-H), 7.51 (d, *J*= 7.2 Hz, 2H, Benzimidazole-H), 7.40-4.17 (s, 2H, -HC-S).;^{13}C-NMR (125 MHz, DMSO-d6): δ169.9, 168.6, 166.4, 152.3, 152.1, 144.0, 140.5, 140.2, 140.1, 139.7, 139.3, 133.6, 132.4, 131.4, 130.7, 129.9, 127.8, 125.0, 124.5, 123.4, 123.3, 123.2, 123.0, 122.4, 121.9, 116.6, 114.7, 114.1, 113.5, 104.3, 34.4. HREI-MS: m/z $[M]^+$calcd for $C_{31}H_{17}N_9O_8S$ 675.0180, found 675.0020.

(3-(((1-(4-hydroxybenzoyl)-1H-benzo[d]imidazol-2-yl)methyl)thio)-8-nitro-5H-[1,2,4]triazino[5,6-b]indol-5-yl)(4-hydroxyphenyl)methanone (12)

^{1}H NMR (500 MHz, DMSO-d_6): δ9.30 (s, 1H, OH), 9.24 (s, 1H, OH),8.79 (s, 1H, Triazinoindole-H), 8.50 (d, *J*= 7.0 Hz, 2H, Ar-H), 8.46 (d, *J* = 8.2 Hz, 2H, Ar-H), 8.42 (d, *J* = 7.8 Hz, 2H, Ar-H), 8.38 (d, *J* = 6.8 Hz, 2H, Ar-H), 8.09 (d, *J* = 7.0 Hz, 1H, Triazinoindole-H), 7.02 (d, *J*= 7.9 Hz, 1H, Triazinoindole-H), 7.53 (d, *J*= 6.9 Hz, 2H, Benzimidazole-H), 7.43 (d, *J*= 8.6 3Hz, 2H, Benzimidazole-H), 4.23 (s, 2H, -HC-S).; ^{13}C-NMR (125 MHz, DMSO-d6): δ 170.9, 167.8, 167.7, 153.6, 153.6, 145.7, 141.3, 141.3, 139.0, 139.0, 138.5, 132.0, 130.3, 130.3, 130.1, 130.1, 126.5, 124.8, 124.8, 123.1, 123.1, 123.4, 123.4, 122.0, 121.7, 115.3, 113.2, 113.1, 111.6, 101.5, 32.6.HREI-MS: m/z $[M]^+$calcd for $C_{31}H_{19}N_7O_6S$ 617.0620, found 617.0531.

(3-((benzo[d]oxazol-2-ylmethyl)thio)-8-nitro-5H-[1,2,4]triazino[5,6-b]indol-5-yl)(4-chlorophenyl)methanone (13)

^{1}H NMR (500 MHz, DMSO-d_6): δ 8.50 (s, 1H, Triazinoindole-H), 8.16 (d, *J*= 7.2 Hz, 1H, Triazinoindole-H), 8.02 (d, *J* =7.5 Hz, 1H, Triazinoindole), 7.65 (d, *J* = 8.2 Hz, 2H, Ar-H), 7.55 (d, *J* = 7.1 Hz, 2H, Ar-H), 7.50 (d, *J* = 8.0 Hz, 2H, Benzooxazole-H), 7.25 (d, *J* = 7.8 Hz, 2H, Benzooxazole-H),; ^{13}C-NMR (125 MHz, DMSO-d6): δ 170.1, 167.4, 152.1, 149.6, 145.0, 141.7, 141.3, 140.0, 132.0, 131.1, 128.8, 128.8, 126.4, 123.2, 124.1, 120.5, 118.6, 113.4, 111.9, 110.4, 101.7, 34.2. HREI-MS: m/z $[M]^+$calcd for $C_{23}H_{13}ClN_6O_4S$ 516.1503, found 516.1494.

methyl 4-(3-((benzo[d]oxazol-2-ylmethyl)thio)-8-nitro-5H-[1,2,4]triazino[5,6-b]indole-5-carbonyl)benzoate (14)

^{1}H NMR (500 MHz, DMSO-d_6δ 8.48 (s, 1H, Triazinoindole-H), 8.13 (d, *J*= 7.7Hz, 1H, Triazinoindole-H), 8.00 (d, *J* =7.3 Hz, 1H, Triazinoindole), 7.61 (d, *J* = 8.0 Hz, 2H, Ar-H), 7.52 (d, *J* = 7.9 Hz, 2H, Ar-H), 7.48 (d, *J* = 7.0 Hz, 2H, Benzooxazole-H), 7.21 (d, *J* = 6.8 Hz, 2H, Benzooxazole-H), 3.50 (s, 3H, -OCH3),; ^{13}C-NMR (125 MHz, DMSO-d6): δ 170.1, 167.4, 165.6, 152.1, 149.6, 145.0, 141.7, 141.3, 140.0, 132.0, 131.1, 128.8, 128.8, 126.4, 123.2, 124.1, 120.5, 118.6, 113.4, 111.9, 110.4, 101.7, 50.3, 34.2. HREI-MS: m/z $[M]^+$calcd for $C_{26}H_{16}N_6O_6S$ 540.1948, found 540.1839.

(3-((benzo[d]oxazol-2-ylmethyl)thio)-8-nitro-5H-[1,2,4]triazino[5,6-b]indol-5-yl)(o-tolyl)methanone (15)

^{1}H NMR (500 MHz, DMSO-d_6): δ 8.41 (s, 1H, Triazinoindole-H), 8.09 (d, *J*= 7.7Hz, 1H, Triazinoindole-H), 8.02 (d, *J* =7.9 Hz, 1H, Triazinoindole), 7.58 (dd, *J* = 7.3, 2.1 Hz, 1H, Ar-H), 7.55-7.51 (m, 1H, Ar-H), 7.48 (dd, *J* = 7.6, 2.0 Hz, 1H, Ar-H), 7.44-7.39 (m, 1H, Ar-H), 7.36 (d, *J* = 8.0 Hz, 2H, Benzooxazole-H), 7.15 (d, *J* = 6.8 Hz, 2H, Benzooxazole-H), 2.15 (s, 3H, CH3),; ^{13}C-NMR (125 MHz, DMSO-d6): δ170.6, 167.7,152.4, 149.5, 145.3, 141.6, 141.2, 140.1, 132.0, 131.5, 128.9, 128.4, 126.7, 123.0, 124.3, 120.7, 118.3, 113.2, 111.8, 110.7, 101.6, 34.1, 20.6. HREI-MS: m/z $[M]^+$calcd for $C_{25}H_{16}N_6O_4S$ 496.1350, found 496.1130.

(3-((benzo[d]oxazol-2-ylmethyl)thio)-8-nitro-5H-[1,2,4]triazino[5,6-b]indol-5-yl)(p-tolyl)methanone (16)

^{1}H NMR (500 MHz, DMSO-d_6): δ 8.39 (s, 1H, Triazinoindole-H), 8.12 (d, *J*= 7.8 Hz, 1H, Triazinoindole-H), 8.00 (d, *J* =8.5 Hz, 1H, Triazinoindole), 7.63 (d, *J* = 8.0 Hz, 2H, Ar-H), 7.52 (d, *J* = 7.7 Hz, 2H, Ar-H), 7.48 (d, *J* = 7.0 Hz,

2H, Benzooxazole-H), 7.23 (d, *J* = 6.8 Hz, 2H, Benzooxazole-H), 2.11 (s, 3H, CH3),; ^{13}C-NMR (125 MHz, DMSO-d6): δ169.1, 166.4, 151.1, 148.6, 144.0, 140.0, 140.3, 139.0, 131.6, 130.1, 127.8, 127.8, 125.4, 122.2, 123.1, 119.5, 117.6, 112.4, 110.9, 109.4, 100.7, 33.2, 20.1. HREI-MS: m/z $[M]^+$calcd for $C_{25}H_{16}N_6O_4S$ 496.1852, found 496.1710.

(3-((benzo[d]oxazol-2-ylmethyl)thio)-8-nitro-5H-[1,2,4]triazino[5,6-b]indol-5-yl)(3-chlorophenyl)methanone (17)

^{1}H NMR (500 MHz, DMSO-d_6): δ 8.48 (s, 1H, Triazinoindole-H),8.19 (d, *J*= 7.7Hz, 1H, Triazinoindole-H), 8.07 (d, *J* =8.9Hz, 1H, Triazinoindole), 7.64 (dd, *J* = 8.3, 2.0Hz, 1H, Ar-H), 7.58 (s, 1H, Ar-H), 7.52 (dd, *J* = 7.3, 2.3Hz, 1H, Ar-H), 7.50 (t, *J* = 6.9Hz, 1H, Ar-H), 7.40 (d, *J* = 8.3Hz, 2H, Benzimidazole-H), 7.21 (d, *J* = 8.4Hz, 2H, Benzimidazole-H),; ^{13}C-NMR (125 MHz, DMSO-d6): δ169.6, 166.7, 159.5, 151.4, 149.5, 146.3, 143.6, 142.2, 141.1, 133.0, 132.5, 130.9, 129.4, 127.7, 125.0, 124.3, 122.7, 120.3, 115.2, 113.8, 111.7, 104.6, 32.1. HREI-MS: m/z $[M]^+$calcd for $C_{24}H_{13}ClN_6O_4S$ 516.4513, found 516.4460.

(3-((benzo[d]oxazol-2-ylmethyl)thio)-8-nitro-5H-[1,2,4]triazino[5,6-b]indol-5-yl)(2-hydroxyphenyl)methanone (18)

^{1}H NMR (500 MHz, DMSO-d_6): δ 9.42 (s, 1H, OH), 8.46 (s, 1H, Triazinoindole-H), 8.14 (d, *J*= 7.3 Hz, 1H, Triazinoindole-H), 8.09 (d, *J* =7.0 Hz, 1H, Triazinoindole), 7.62 (dd, *J* = 8.3, 2.1 Hz, 1H, Ar-H), 7.59-7.55 (m, 1H, Ar-H), 7.52 (dd, *J* = 7.6, 2.0 Hz, 1H, Ar-H), 7.49-7.46 (m, 1H, Ar-H), 7.42 (d, *J* = 7.0 Hz, 2H, Benzooxazole-H), 7.25 (d, *J* = 8.8 Hz, 2H, Benzooxazole-H),; ^{13}C-NMR (125 MHz, DMSO-d6): δ 171.6, 169.7, 155.4, 151.5, 149.3, 145.6, 143.2, 142.1, 141.0, 134.5, 130.9, 129.4, 128.7, 125.0, 124.3, 122.7, 121.3, 115.2, 116.8, 112.7, 104.6, 35.1. HREI-MS: m/z $[M]^+$calcd for $C_{24}H_{14}N_6O_5S$ 498.3842, found 498.3735.

(3-((benzo[d]oxazol-2-ylmethyl)thio)-8-nitro-5H-[1,2,4]triazino[5,6-b]indol-5-yl)(m-tolyl)methanone (19)

^{1}H NMR (500 MHz, DMSO-d_6δ 8.39 (s, 1H, Triazinoindole-H), 8.06 (d, *J*= 8.7 Hz, 1H, Triazinoindole-H), 8.00 (d, *J* =6.9 Hz, 1H, Triazinoindole), 7.50 (dd, *J* = 8.3, 2.3 Hz, 1H, Ar-H), 7.45 (s, 1H, Ar-H), 7.41 (dd, *J* = 7.2, 2.0 Hz, 1H, Ar-H), 7.38 (t, *J* = 6.7 Hz, 1H, Ar-H), 7.35 (d, *J* = 7.3 Hz, 2H, Benzooxazole-H), 7.15 (d, *J* = 8.4 Hz, 2H, Benzooxazole-H), 2.11 (s, 3H, CH3),; ^{13}C-NMR (125 MHz, DMSO-d6): δ 169.6, 166.7, 164.4, 163.0, 151.4, 149.5,146.3, 143.6, 142.2, 141.1, 133.0, 132.5, 130.9, 129.4, 127.7, 125.0, 124.3, 122.7, 120.3, 115.2, 113.8, 111.7, 104.6, 32.1, 20.6. HREI-MS: m/z $[M]^+$calcd for $C_{25}H_{16}N_6O_4S$ 496.1950, found 496.1843.

(3-((benzo[d]oxazol-2-ylmethyl)thio)-8-nitro-5H-[1,2,4]triazino[5,6-b]indol-5-yl)(3-hydroxy-4-methoxyphenyl)methanone (20)

^{1}H NMR (500 MHz, DMSO-d_6): δ 9.55 (s, 1H, OH), 8.51 (s, 1H, Triazinoindole-H), 8.19 (d, *J*= 8.7 Hz, 1H, Triazinoindole-H), 8.10 (d, *J* =7.4 Hz, 1H, Triazinoindole), 7.69 (d, *J* = 8.0 Hz, 1H, Ar-H), 7.57 (d, *J* = 7.6 Hz, 1H, Ar-H), 7.54 (s, 1H, Ar-H), 7.57 (d, *J* = 7.6 Hz, 1H, Ar-H), 7.51 (d, *J* = 7.0 Hz, 2H, Benzooxazole-H), 7.35 (d, *J* = 8.8 Hz, 2H, Benzooxazole-H), 3.60 (s, 3H, -OCH3),; ^{13}C-NMR (125 MHz, DMSO-d6): δ 172.1, 168.4, 166.6, 154.1,

150.6, 146.0, 142.7, 142.3, 141.0, 134.0, 133.1, 129.8, 128.6, 127.4, 125.2, 124.1, 123.5, 119.6, 114.4, 112.9, 110.4, 109.4, 104.7, 35.2, 21.4. HREI-MS: m/z $[M]^+$calcd for $C_{25}H_{16}N_6O_6S$ 528.2913, found 528.2801.

(3-((benzo[d]oxazol-2-ylmethyl)thio)-8-nitro-5H-[1,2,4]triazino[5,6-b]indol-5-yl)(2,4-dimethoxyphenyl)methanone (21)

^{1}H NMR (500 MHz, DMSO-d_6): δ 8.48 (s, 1H, Triazinoindole-H), 8.15 (d, *J*= 8.7 Hz, 1H, Triazinoindole-H), 8.07 (d, *J* =7.4 Hz, 1H, Triazinoindole), 7.62 (d, *J* = 7.4 Hz, 1H, Ar-H), 7.54 (d, *J* = 8.6 Hz, 1H, Ar-H), 7.51 (s, 1H, Ar-H), 7.49 (d, *J* = 6.6 Hz, 1H, Ar-H), 7.46 (d, *J* = 7.4 Hz, 2H, Benzooxazole-H), 7.30 (d, *J* = 7.8 Hz, 2H, Benzooxazole-H), 3.57 (s, 3H, -OCH3), 3.51 (s, 3H, -OCH3),; ^{13}C-NMR (125 MHz, DMSO-d6): δ 171.4, 167.2, 166.3, 154.3, 150.4, 146.1, 142.6, 142.2, 141.7, 134.9, 133.5, 129.5, 128.3, 127.2, 125.7, 124.2, 123.4, 119.4, 114.3, 112.6, 110.2, 109.0, 104.5, 35.0, 21.4, 21.0. HREI-MS: m/z $[M]^+$calcd for $C_{26}H_{18}N_6O_6S$ 542.1425, found 542.1320.

(3-((benzo[d]oxazol-2-ylmethyl)thio)-8-nitro-5H-[1,2,4]triazino[5,6-b]indol-5-yl)(2,3-dihydroxyphenyl)methanone (22)

^{1}H NMR (500 MHz, DMSO-d_6): δ9.60 (s, 1H, OH), 9.48 (s, 1H, OH), 8.60 (s, 1H, Triazinoindole-H), 8.30 (d, *J*= 6.7 Hz, 1H, Triazinoindole-H), 8.25 (d, *J* =7.9 Hz, 1H, Triazinoindole), 7.70 (dd, *J* = 8.0, 2.3 Hz, 1H, Ar-H), 7.67 (dd, *J* = 7.4, 2.0 Hz, 1H, Ar-H), 7.55 (t, *J* = 7.7 Hz, 1H, Ar-H), 7.45 (d, *J* = 7.3 Hz, 2H, Benzooxazole-H), 7.30 (d, *J* = 8.3 Hz, 2H, Benzooxazole-H),; ^{13}C-NMR (125 MHz, DMSO-d6): δ 172.6, 168.7, 166.4, 165.0, 156.4, 153.5,49.3, 145.6, 143.2, 141.9, 135.0, 133.5, 132.9, 130.4, 129.7, 127.0, 125.3, 124.7, 122.3, 117.2, 114.8, 113.7, 106.6, 35.1. HREI-MS: m/z $[M]^+$calcd for $C_{24}H_{14}N_6O_6S$ 514.7793, found 514.7687.

(3-((benzo[d]oxazol-2-ylmethyl)thio)-8-nitro-5H-[1,2,4]triazino[5,6-b]indol-5-yl)(2-fluorophenyl)methanone (23)

^{1}H NMR (500 MHz, DMSO-d_6): δ 8.50 (s, 1H, Triazinoindole-H), 8.17 (d, *J*= 7.6Hz, 1H, Triazinoindole-H), 8.12 (d, *J* =8.0 Hz, 1H, Triazinoindole), 7.67 (dd, *J* = 8.0, 1.8 Hz, 1H, Ar-H), 7.64-7.61 (m, 1H, Ar-H), 7.58 (dd, *J* = 8.6, 1.7 Hz, 1H, Ar-H), 7.55-7.51 (m, 1H, Ar-H), 7.49 (d, *J* = 7.9 Hz, 2H, Benzoxazole-H), 7.35 (d, *J* = 7.5 Hz, 2H, Benzoxazole-H),; ^{13}C-NMR (125 MHz, DMSO-d6): δ 171.9, 169.6, 155.7, 151.4, 149.8, 145.2, 143.6, 142.0, 141.3, 134.7, 130.9, 129.3, 128.6, 125.6, 124.7, 122.5, 121.0, 115.1, 116.9, 112.6, 104.3, 35.5. HREI-MS: m/z $[M]^+$calcd for $C_{24}H_{13}FN_6O_4S$ 500.0801, found 500.0795.

(3-((benzo[d]oxazol-2-ylmethyl)thio)-8-nitro-5H-[1,2,4]triazino[5,6-b]indol-5-yl)(3-fluorophenyl)methanone (24)

^{1}H NMR (500 MHz, DMSO-d_6): δ 8.47 (s, 1H, Triazinoindole-H), 8.15 (d, *J*= 7.0 Hz, 1H, Triazinoindole-H), 8.10 (d, *J* =8.1 Hz, 1H, Triazinoindole), 7.65 (dd, *J* = 7.0, 1.9 Hz, 1H, Ar-H), 7.61 (t, *J* = 7.7 Hz, 1H, Ar-H), 7.56 (dd, *J* = 8.3, 2.0 Hz, 1H, Ar-H), 7.53 (s, 1H, Ar-H), 7.46 (d, *J* = 7.4 Hz, 2H, Benzooxazole-H), 7.33 (d, *J* = 8.5 Hz, 2H, Benzooxazole-H),; ^{13}C-NMR (125 MHz, DMSO-d6): δ 171.9, 169.5, 155.6, 151.3, 149.7, 145.1, 143.5, 142.3, 141.2,

134.6, 130.8, 129.2, 128.5, 125.5, 124.6, 122.4, 121.3, 115.0, 116.8, 112.5, 104.2, 35.4. HREI-MS: m/z $[M]^+$calcd for $C_{24}H_{13}FN_6O_4S$ 500.0701, found 500.0695.

(3-((benzo[d]oxazol-2-ylmethyl)thio)-8-nitro-5H-[1,2,4]triazino[5,6-b]indol-5-yl)(3-nitrophenyl)methanone (25)

^{1}H NMR (500 MHz, DMSO-d_6): δ 8.75 (s, 1H, Ar-H), 8.64 (s, 1H, Triazinoindole-H), 8.27 (t, *J* = 7.2 Hz, 1H, Ar-H), 8.09-8.01 (m, 1H, Ar-H), 8.00 (d, *J* = 7.3Hz, 1H, Triazinoindole-H), 7.62-7.51 (m, 1H, Ar-H), 7.50 (d, *J* = 6.8 Hz, 2H, Benzooxazole-H), 7.07 (dd, *J* = 7.2 Hz, 1H, Triazinoindole-H), 6.45 (d, *J* = 7.2 Hz, 2H, Benzooxazole -H),; ^{13}C-NMR (125 MHz, DMSO-d6): δ 168.0, 148.8, 141.2, 134.9, 130.6, 130.2, 129.7, 129.6, 129.4, 129.0, 128.2, 127.5, 127.3, 127.0, 126.5, 126.4, 125.8, 124.3, 124.9, 121.3, 120.0, 118.3, 109.9, 40.0. HREI-MS: m/z $[M]^+$calcd for $C_{24}H_{13}N_7O_6S$ 527.4513, found 527.4460.

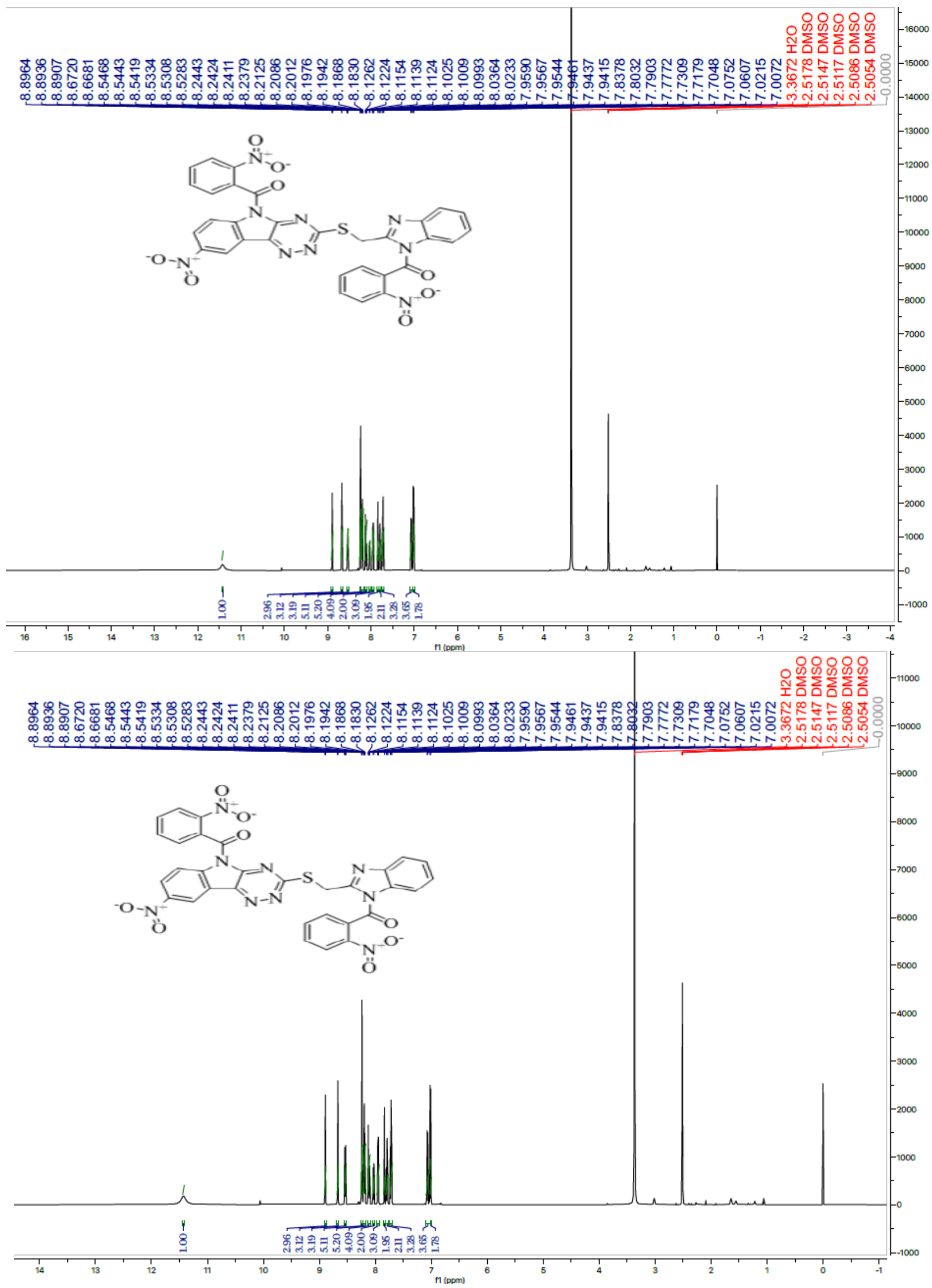

Figure S2. Low resolution proton NMR spectrum of (8-nitro-3-(((1-(2-nitrobenzoyl)-1H-benzo[d]imidazol-2-yl)methyl)thio)-5H-[1,2,4]triazino[5,6-b]indol-5-yl)(2-nitrophenyl)methanone (10)

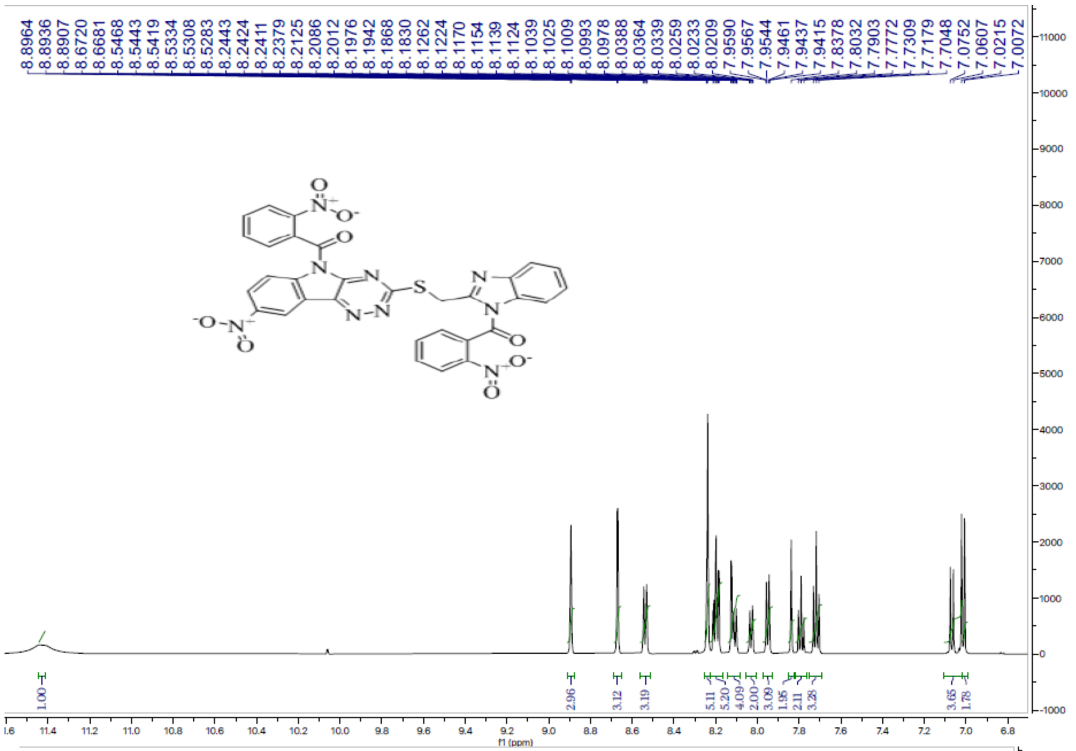
8.8964 8.8936 8.8907 8.6720 8.6681 8.5468 8.5443 8.5419 8.5334 8.5308 8.5283 8.2443 8.2424 8.2411 8.2379 8.2125 8.2086 8.2012 8.1976 8.1942 8.1868 8.1830 8.1262 8.1224 8.1170 8.1154 8.1139 8.1124 8.1039 8.1025 8.1009 8.0993 8.0978 8.0388 8.0364 8.0339 8.0259 8.0233 8.0209 7.9590 7.9567 7.9544 7.9461 7.9437 7.9415 7.8378 7.8032 7.7903 7.7772 7.7309 7.7179 7.7048 7.0752 7.0607 7.0215 7.0072
1.00 2.96 3.12 3.19 5.11 5.20 4.09 2.00 3.09 1.95 2.11 3.28 3.65 1.78
f1 (ppm)

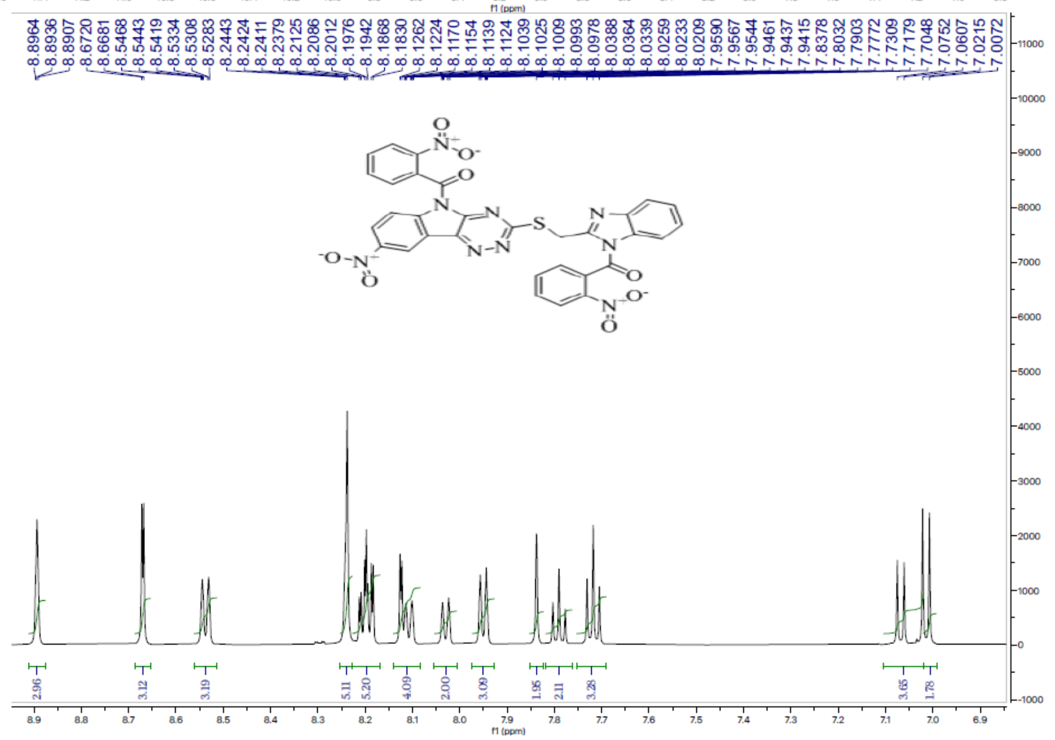
8.8964 8.8936 8.8907 8.6720 8.6681 8.5468 8.5443 8.5419 8.5334 8.5308 8.5283 8.2443 8.2424 8.2411 8.2379 8.2125 8.2086 8.2012 8.1976 8.1942 8.1868 8.1830 8.1262 8.1224 8.1170 8.1154 8.1139 8.1124 8.1039 8.1025 8.1009 8.0993 8.0978 8.0388 8.0364 8.0339 8.0259 8.0233 8.0209 7.9590 7.9567 7.9544 7.9461 7.9437 7.9415 7.8378 7.8032 7.7903 7.7772 7.7309 7.7179 7.7048 7.0752 7.0607 7.0215 7.0072
2.96 3.12 3.19 5.11 5.20 4.09 2.00 3.09 1.95 2.11 3.28 3.65 1.78
f1 (ppm)

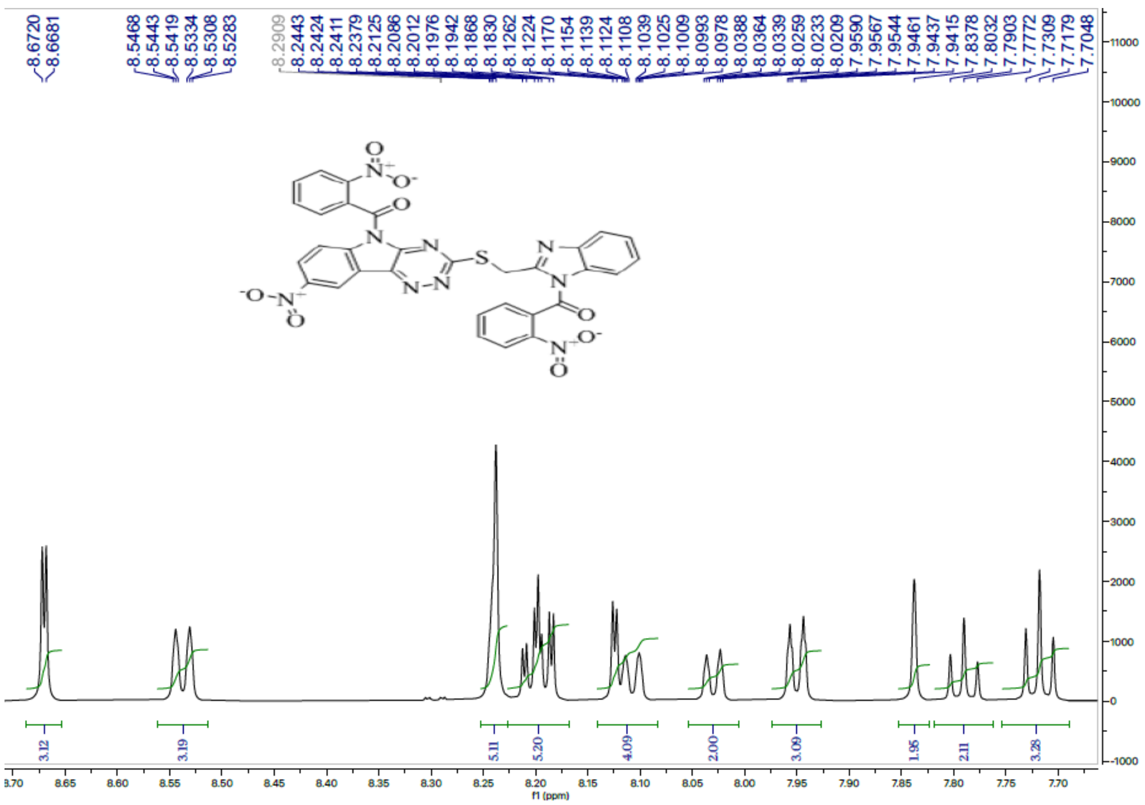

Figure S3. High resolution proton NMR spectrum of (8-nitro-3-(((1-(2-nitrobenzoyl)-1H-benzo[d]imidazol-2-yl)methyl)thio)-5H-[1,2,4]triazino[5,6-b]indol-5-yl)(2-nitrophenyl)methanone (10)

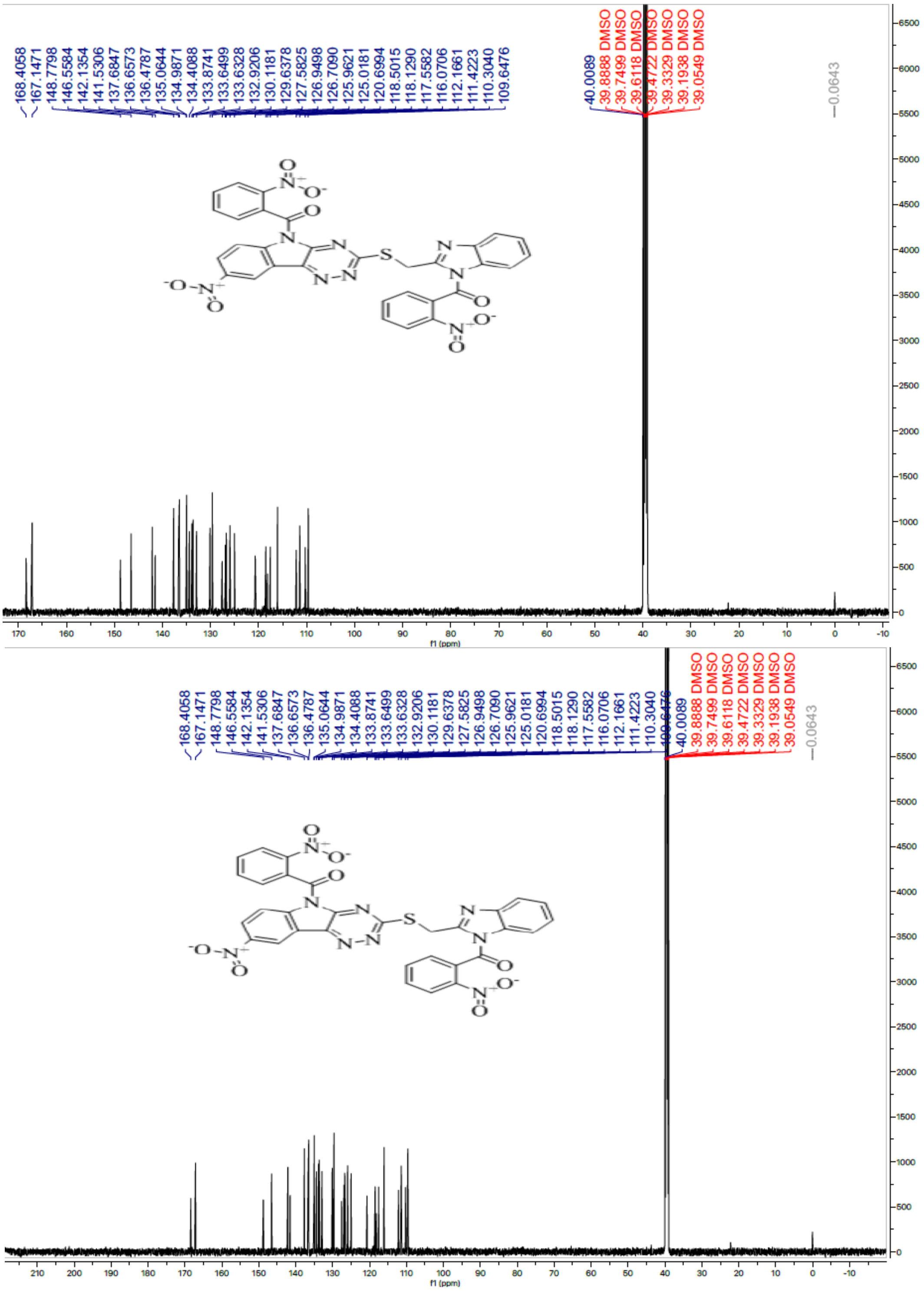

Figure S4. Low resolution ^{13}C-NMR spectrum of (8-nitro-3-(((1-(2-nitrobenzoyl)-1H-benzo[d]imidazol-2-yl)methyl)thio)-5H-[1,2,4]triazino[5,6-b]indol-5-yl)(2-nitrophenyl)methanone (10)

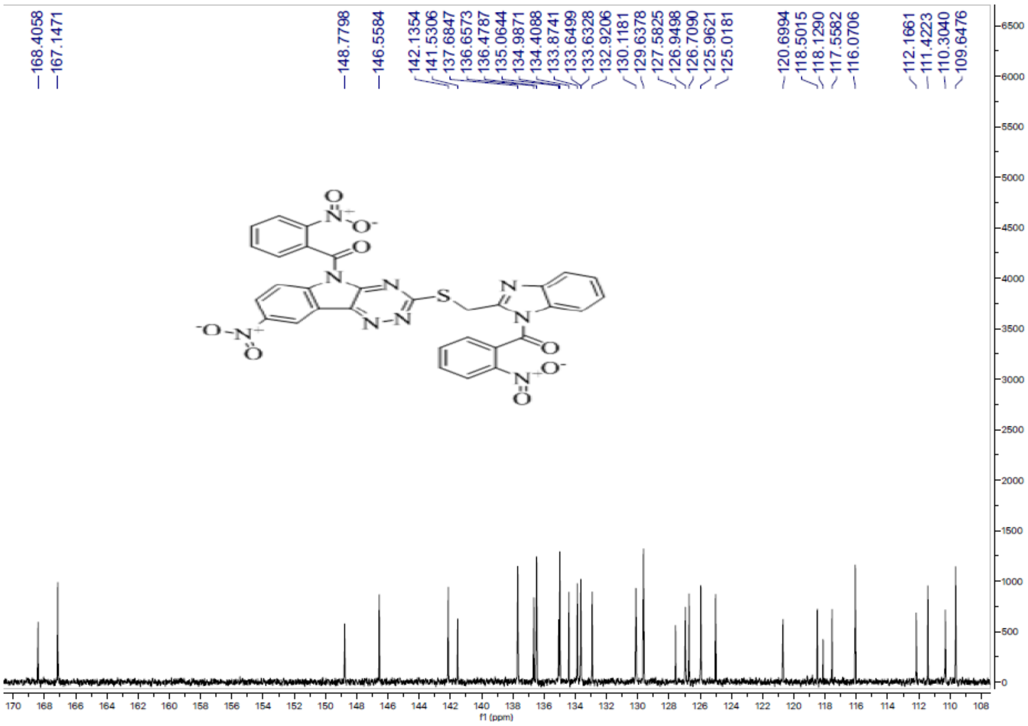

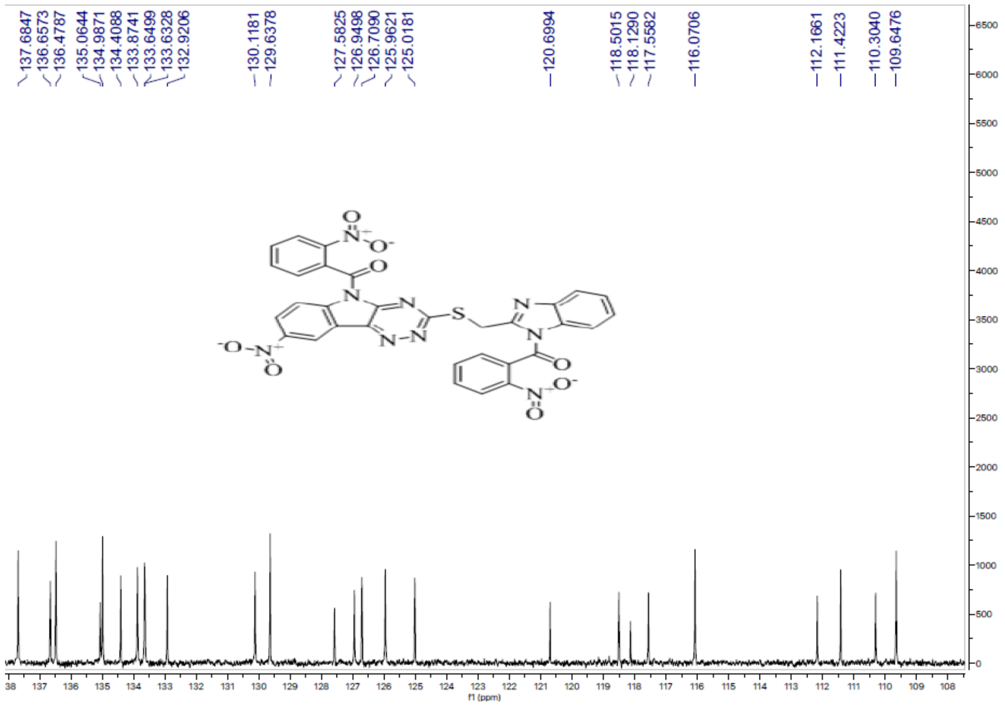

Figure S5. High resolution ^{13}C-NMR spectrum of (8-nitro-3-(((1-(2-nitrobenzoyl)-1H-benzo[d]imidazol-2-yl)methyl)thio)-5H-[1,2,4]triazino[5,6-b]indol-5-yl)(2-nitrophenyl)methanone (10)

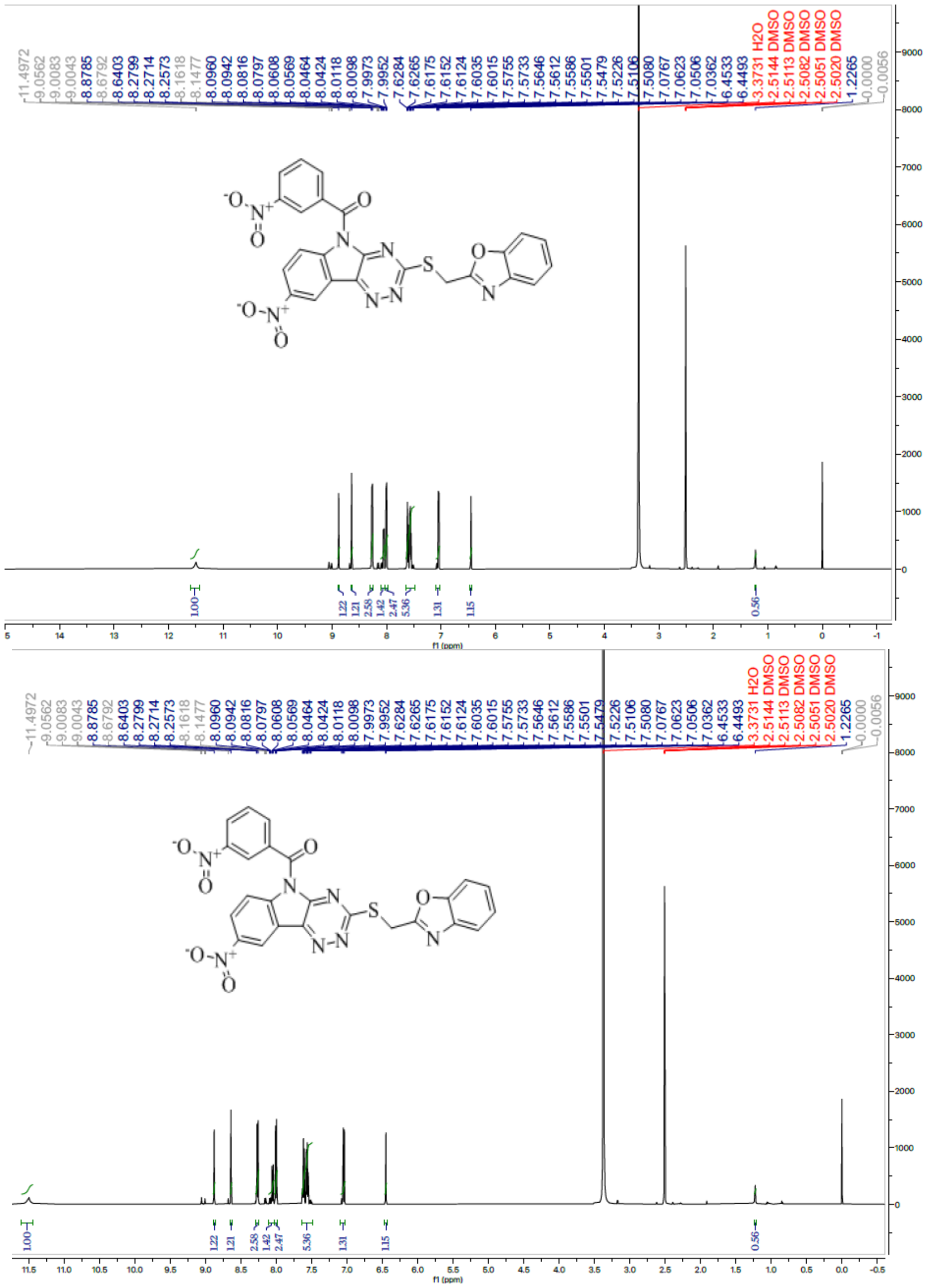

Figure S6. Low resolution proton NMR spectrum of (3-((benzo[d]oxazol-2-ylmethyl)thio)-8-nitro-5H-[1,2,4]triazino[5,6-b]indol-5-yl)(3-nitrophenyl)methanone (25)

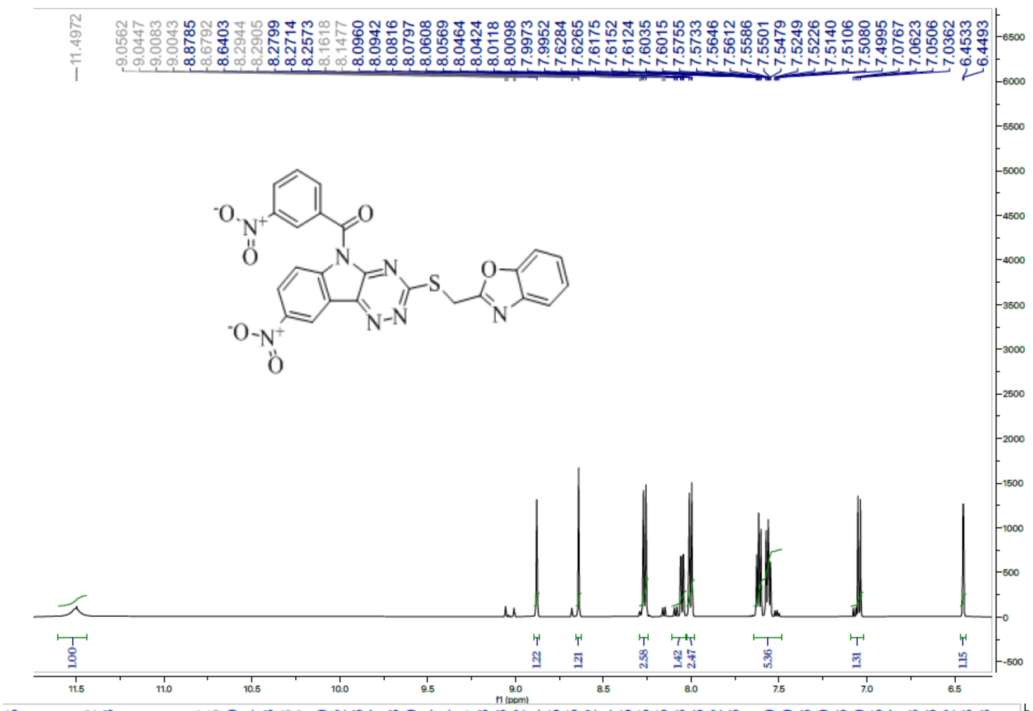

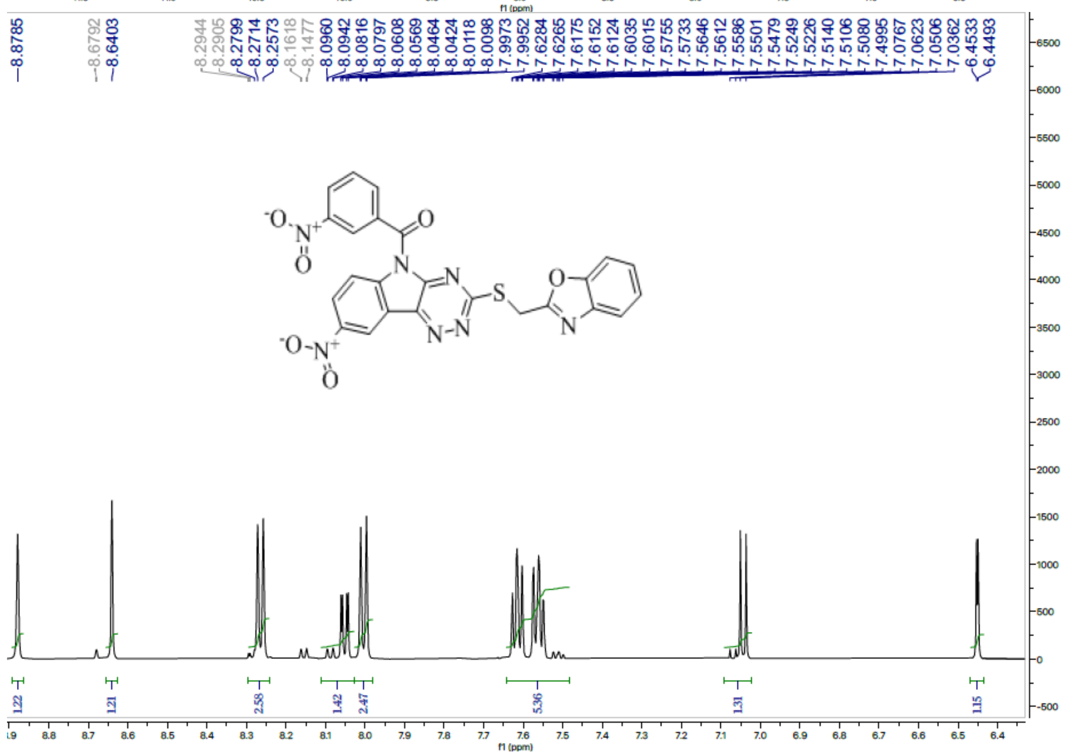

Figure S7. High resolution proton NMR spectrum of (3-((benzo[d]oxazol-2-ylmethyl)thio)-8-nitro-5H-[1,2,4]triazino[5,6-b]indol-5-yl)(3-nitrophenyl)methanone (25)

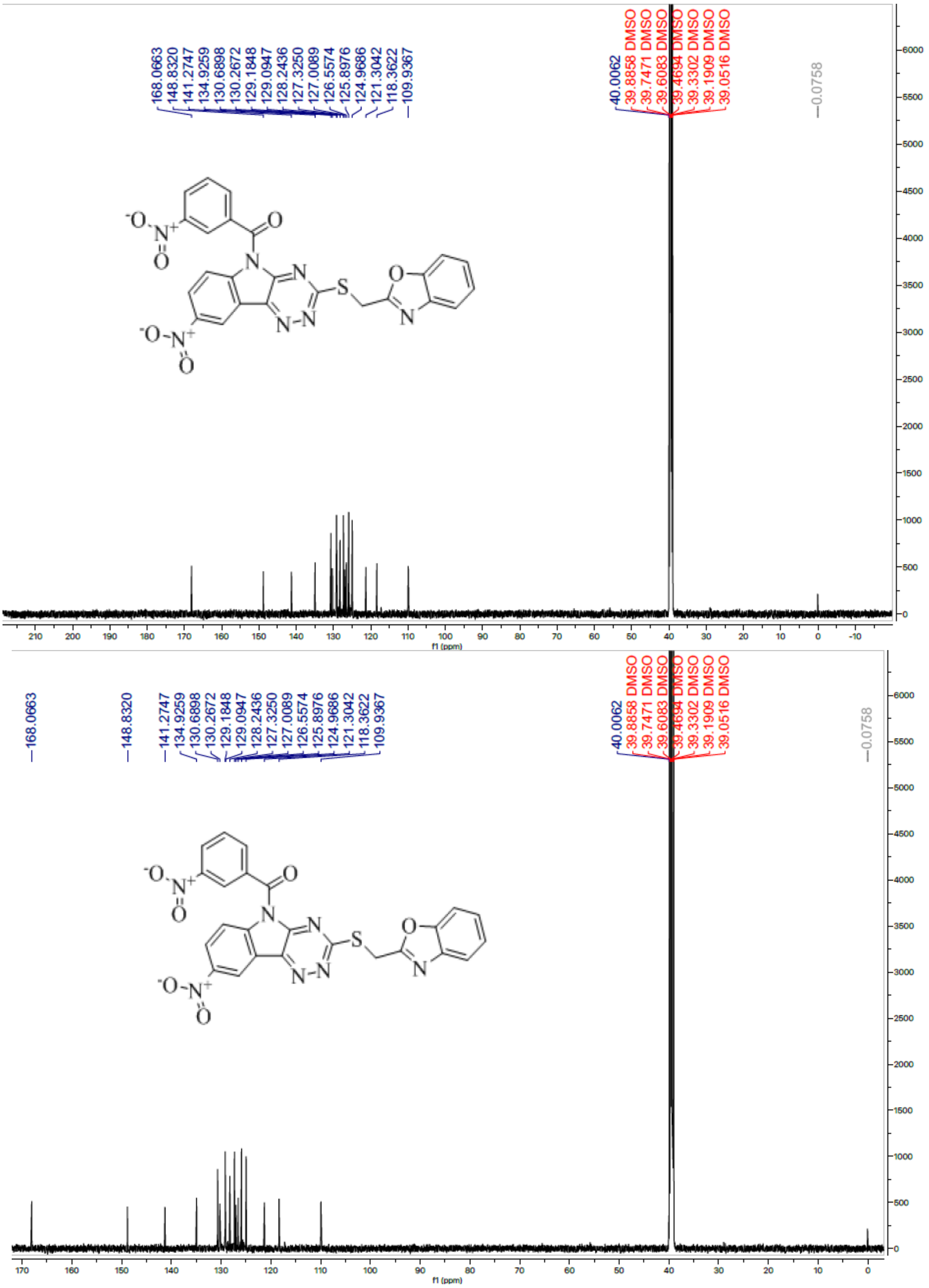

Figure S8. Low resolution 13C-NMR spectrum of (3-((benzo[d]oxazol-2-ylmethyl)thio)-8-nitro-5H-[1,2,4]triazino[5,6-b]indol-5-yl)(3-nitrophenyl)methanone (25)

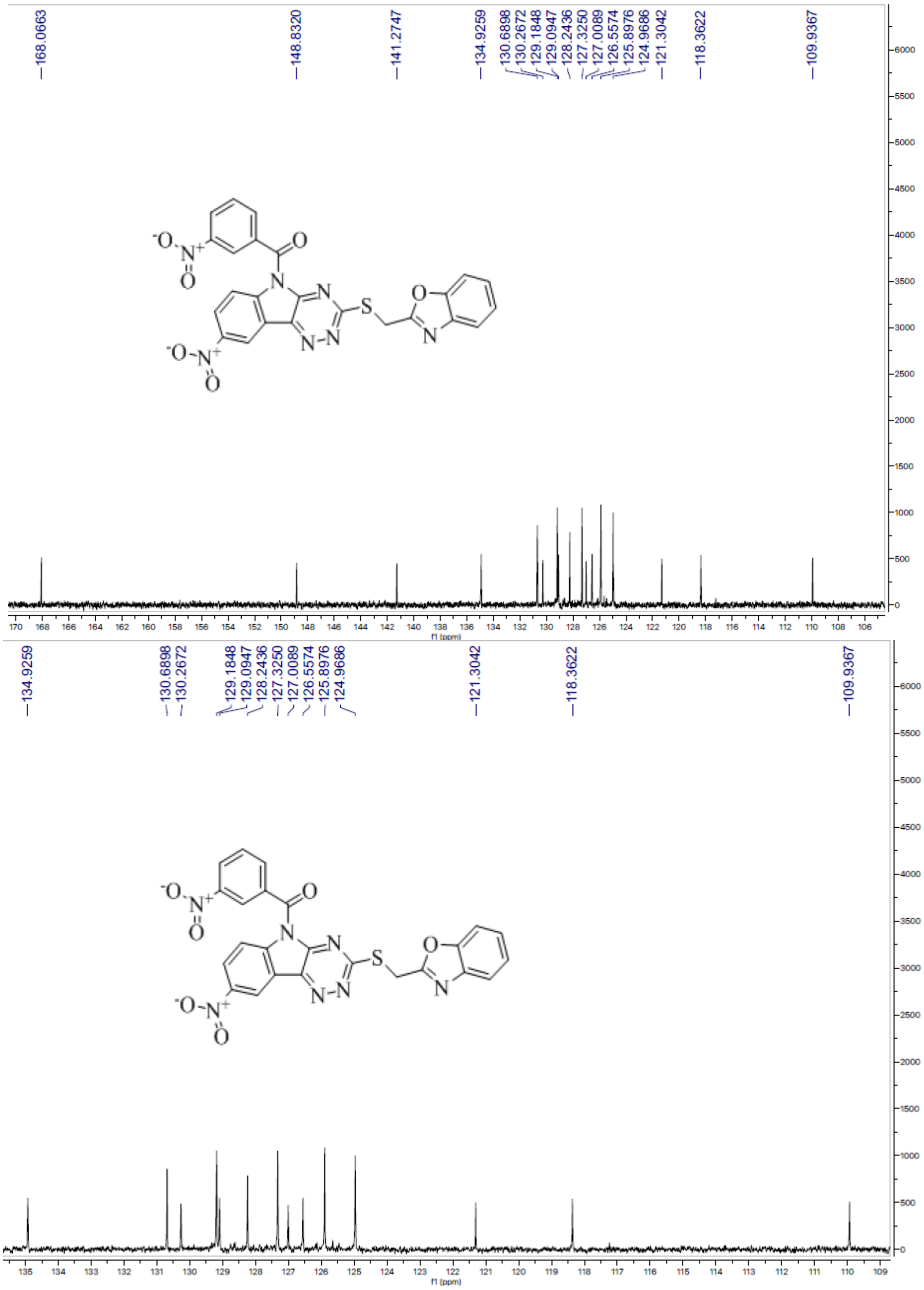

Figure S9. High resolution 13C-NMR spectrum of (3-((benzo[d]oxazol-2-ylmethyl)thio)-8-nitro-5H-[1,2,4]triazino[5,6-b]indol-5-yl)(3-nitrophenyl)methanone (25)
